# Changes in the gut microbiota of forest musk deer (*Moschus berezovskii*) during *ex situ* conservation

**DOI:** 10.3389/fmicb.2022.969593

**Published:** 2022-09-08

**Authors:** Yuanlin Jiang, Xiangyu Han, Mengqi Li, Nuannuan Feng, Pengcheng Yang, Haoxi Zhao, Chenxi Zhang, Minghui Shi, Zhixin Huang, Rubin Sun, Shuqiang Liu, Defu Hu

**Affiliations:** ^1^School of Ecology and Nature Conservation, Beijing Forestry University, Beijing, China; ^2^Zhangzhou Pien Tze Huang Pharmaceutical Co., Ltd., Zhangzhou, Fujian, China; ^3^Huailai Zhiyangtianbao Technical Development Co., Ltd., Zhangjiakou, China

**Keywords:** forest musk deer, gut microbiota, artificial breeding, *ex situ*, food

## Abstract

*Ex situ* conservation is an important technique for protecting rare and endangered wildlife, and maintaining stable individual health is crucial to its success. Gut microbiota composition is a critical indicator of animal health and should therefore be closely monitored during *ex situ* conservation to track impacts on animal health. Forest musk deer (*Moschus berezovskii*) were historically distributed in Hebei Province, China, however, they are now extinct in the region. Thus, *ex situ* conservation efforts were conducted in 2016 whereby approximately 50 individuals were artificially migrated from Weinan, Shaanxi to Huailai, Hebei. To monitor gut health of these migrated individuals, we used 16S rRNA high-throughput sequencing technology to examine the microbiota differences between Huailai juvenile and Weinan juvenile groups, and between Huailai adult and Weinan adult groups. Alpha diversity analysis indicated that the richness of microbiota significantly decreased after migration to the Huailai area, and the beta diversity results also showed significant dissimilarity in gut microbial communities, demonstrating the distinct microbial structure differences in the forest musk deer population from the two areas, for both juvenile and adult groups, respectively. In addition, PICRUSt functional profile prediction indicated that the functions of gut digestion and absorption, and degradation of toxic substances were significantly weakened after *ex situ* conservation. Differences in diet composition between the individuals of the two sites were also observed and the impact of food on gut microbiota compositions within forest musk deer during *ex situ* conservation was investigated. This study provides a theoretical basis for developing *ex situ* conservation measures, especially for the protection of forest musk deer.

## Introduction

Forest musk deer (*Moschus berezovskii*) were widely distributed in China; however, the wild population rapidly declined from 2.5 million in the 1950s to less than 100,000 in the 1990s due to indiscriminate poaching and reduced natural habitat areas (Jie et al., 2015; Cai et al., 2020). Since population expansion by *ex situ* artificial breeding followed by reintroduction to the wild has shown to be effective in conserving endangered animals, China started breeding forest musk deer in 1958 to maintain sufficient captive-bred deer for reintroduction, which helped restore the forest musk deer population for valuable musk harvests while reducing the pressure on wild musk deer resources (He et al., 2014).

Promoting *ex situ* artificial breeding is the first step toward protecting this species. The successful artificial breeding of forest musk deer in China was mainly concentrated in Shaanxi and Sichuan Provinces. A survey indicated that the population of forest musk deer exceeded 30,000 individuals in 2013 (Feng et al., 2013), and its partial historical distribution area was restored. Hebei Province was also the historical distribution area of musk deer, however, over-hunting and habitat destruction resulted in population extinction (Wen, 2016). We conducted *ex situ* breeding of forest musk deer in the area in order to restore the distribution of musk deer in Hebei Province.

Focusing on health status is critical during the process of *ex situ* conservation of forest musk deer. Gut microbiota is an important physiological indicator of animal health; its homeostasis is integral to maintain various health related functions (Prakash et al., 2011; Aziz et al., 2013). An imbalance in intestinal microbiota is often accompanied by a reduction in microbiota diversity. The decreased diversity and richness of animal gut microbiota can increase the probability of diseases in the affected animals (Li et al., 2018; Zhang et al., 2019; Mao et al., 2020; Zhao et al., 2021). While gut microbiota is influenced by a variety of factors, food is the most direct factor affecting the diversity of animal intestinal microbiota (Hicks et al., 2018). Food is a substrate for microbial fermentation and drives the composition and metabolism of microbial communities (Brian et al., 2011). In addition, it is a direct influence on intestinal microbiota and a key factor in the co-evolution of animals and their intestinal microbiota (Nicholson et al., 2012; Yang et al., 2014).

This study analyzed and compared differences in the gut microbiota of forest musk deer in the immigration (Huailai, Hebei Province) and emigration areas (Weinan, Shaanxi Province) to increase understanding of composition changes in their gut microbiota during *ex situ* conservation and provide a scientific reference to improve and evaluate their health status. The study is beneficial for accumulating *ex situ* conservation experience and improving the success rate of *ex situ* conservation of breeding musk deer.

## Materials and methods

### Basic information on research areas

The geographical information of the Weinan, Shaanxi and Huailai, Hebei forest musk deer breeding bases and food comparison is outlined in Supplementary Table 1 and Table 1, respectively. The Hebei forest musk deer population artificially migrated from the Shaanxi forest in 2016. Since then, the population size has increased to more than 200 individuals due to the initial success of the artificial breeding programs.

**TABLE 1:** Food comparison between Weinan and Huailai areas.

Food category	Weinan	Huailai
Roughage forage	Siberian elm leaves and paper mulberry leaves	Siberian elm leaves, apricot leaves, and mulberry leaves
Succulent feed	Pumpkins, carrots, dandelions, and apples	Pumpkin, carrot, white radish, and apple
Concentrate	Sesame, peanut, black bean, soya bean, wheat bran, and corn	Sesame, peanut, soya bean, wheat bran, and corn
Traditional Chinese medicine	Dang Shen (Radix Codonopsis), Bai Zhu (Rhizoma Atractylodis Macrocephalae), Fu Ling (Poria), Gan Cao (Radix Glycyrrhizae), Sheng Shan Zha (Raw Fructus Crataegi), Ji Nei Jin (Endothelium Corneum Gigeriae Galli), Chen Pi (Pericarpium Citri Reticulatae), Mai Ya (Fructus Hordei Germinatus), Shen Qu (Massa Medicata Fermentata), Huang Qi (Radix Astragali), Cao Dou Kou (Semen Alpiniae Katsumadai), Hou Po (Cortex Magnoliae Officinalis), Bai Kou (Fructus Amomi Rotundus), Jin Yin Hua (Flos Lonicerae), Lian Qiao (Fructus Forsythiae), Guan Zhong (Rhizoma Cyrtomii), Chai Hu (Radix Bupleuri), and Ban Lan Gen (Radix Isatidis)	None

Weinan is in the middle reaches of the Yellow River in the eastern Guanzhong Plain in Shaanxi Province. It has a warm, temperate, semi-humid, and semi-arid monsoon climate, with four distinct seasons, sufficient light, and suitable rainfall; its specific environmental and food conditions are shown in Supplementary Table 1 and Table 1, respectively.

Huailai is in the northwest of Hebei Province in the temperate semi-arid region and has a temperate continental monsoon climate. It has four distinct seasons, sufficient light, hot and rainy seasons similar to Weinan, and a large temperature difference between day and night. The summer weather is warm and humid, with increased precipitation. In winter, cold air activity is frequent, and the weather is cold and snowy. The specific environmental and food conditions are shown in Supplementary Table 1 and Table 1, respectively.

### Traditional Chinese medicine formula and feeding method

The wild forest musk deer are known to forage freely with many plants in its daily diet classified as traditional Chinese medicine (TCM), which have been extensively studied as a safe, non-toxic or less toxic dietary additive in animal production practices (Yeh et al., 2011; Gong et al., 2014). Therefore, the TCM was added to their diet to compensate for the lack of nutrients in artificial feed during the breeding process in Weinan, Shaanxi (Yang et al., 2011).

In this study, the TCM was orally administered as a dietary supplement to the forest musk deer. A mixture of 18 different TCMs was ground into powder and added to the feed of adult and juvenile forest musk deer in the Weinan area of Shaanxi. While 5 g of the TCM were administered twice daily for these forest musk deer, none were given to those in the Huailai area of Hebei Province.

The main ingredients of the TCMs include Dang Shen (Radix Codonopsis) 200 g, Bai Zhu (Rhizoma Atractylodis Macrocephalae) 100 g, Fu Ling (Poria) 60 g, Gan Cao (Radix Glycyrrhizae) 50 g, Sheng Shan Zha (Raw Fructus Crataegi) 200 g, Ji Nei Jin (Endothelium Corneum Gigeriae Galli) 100 g, Chen Pi (Pericarpium Citri Reticulatae) 80 g, Mai Ya (Fructus Hordei Germinatus) 100 g, Shen Qu (Massa Medicata Fermentata) 50 g, Huang Qi (Radix Astragali) 200 g, Cao Dou Kou (Semen Alpiniae Katsumadai) 50 g, Hou Po (Cortex Magnoliae Officinalis) 100 g, and Bai Kou (Fructus Amomi Rotundus) 50 g; Cold prevention ingredients: Jin Yin Hua (Flos Lonicerae) 200 g, Lian Qiao (Fructus Forsythiae) 200 g, Guan Zhong (Rhizoma Cyrtomii) 200 g, Chai Hu (Radix Bupleuri) 200 g, and Ban Lan Gen (Radix Isatidis) 200 g.

### Animals and sample collection

Twelve healthy adult forest musk deer (6 male, 6 female; 4–8 years old) and 12 healthy juvenile forest musk deer (6 male, 6 female; 1–2 years old) were selected from the Weinan and Huailai areas in mid-December 2021 (the adult deer that were migrated to Hebei Province were brought as adults at Shaanxi Province). The deer were divided into two juvenile groups, Huailai juvenile (HJ) and Weinan juvenile (WJ), and two adult groups, Huailai adult (HA) and Weinan adult (WA).

Fresh fecal samples were collected in the early morning from previously cleaned holding areas. Disposable sterile gloves were worn during sample collection to avoid human contamination. Samples were stored in sterile centrifuge tubes immediately after collection and sealed to avoid cross-contamination. Immediately after sampling, the fecal samples were stored in liquid nitrogen at −80°C until DNA extraction.

### DNA extraction, polymerase chain reaction, and paired-end sequencing

Total DNA was extracted with the QIAamp DNA Stool Mini Kit (QIAGEN, Hilden, Germany) according to the manufacturer’s protocol. The integrity of the nucleic acids were determined visually by electrophoresis on a 1.0% agarose gel containing ethidium bromide. The concentration and purity of each DNA extract were determined using a Qubit dsDNA HS Assay Kit (Life Technologies, Carlsbad, CA, United States). The extracted total DNA was preserved at −80°C.

After the total DNA of the sample was extracted, the primers were designed according to the conserved region. The V3–V4 region of the bacterial 16S- rRNA gene was amplified by PCR (95°C for 5 min, followed by 25 cycles of 95°C for 30 s, 50°C for 30 s, 72°C for 40 s, and 72°C for 7 min) using the primers 338F (5′-ACTCCTACGGGAGGCAGCA-3′) and 806R (5′-GGACTACHVGGGTWTCTAAT-3) and the sequencing joint was added at the end of the primers. The PCR products were mixed with the 2 × loading buffer and were subjected to 1.8% agarose gel electrophoresis for detection. The products were purified, quantified, and homogenized to form a sequencing library. The constructed library was subjected to library quality inspection by Qsep-400, and the qualified library was sequenced by Illumina Novaseq 6000. The original image data file obtained by high throughput sequencing was transformed into Sequenced Reads by Base Calling analysis.

### Data analysis

Trimmomatic v0.33 (Bolger et al., 2014) was used to filter the raw sequenced reads. The primer sequences were identified and removed using Cutadapt 1.9.1 (Martin, 2011) to obtain clean reads. Subsequently, Usearch v10 (Magoc and Salzberg, 2011) was employed to concatenate the clean reads of each sample using the overlap command, and the concatenated data were filtered by length, according to the range of sequence lengths for the different regions. To obtain the final effective reads, chimeric sequences were identified and removed using UCHIME v4.2 (Edgar et al., 2011). Information analysis included feature classification operational taxonomic units (OTUs) and amplicon sequence variants (ASVs), diversity, difference, correlation, and function prediction analyses.

Clustering was performed using Usearch for reads with 97% similarity to obtain OTUs. Using SILVA (Quast et al., 2013) as the reference database, a Naïve Bayes classifier was used to perform taxonomic annotations of the feature sequences, to obtain the taxonomic information corresponding to each feature needed to determine the community composition of each sample at each taxonomic rank (phylum, class, order, family, genus, and species). QIIME (Bolyen et al., 2019) was used to generate species abundance tables at different taxonomic ranks; R functions were then employed to create community structure maps for the samples at each taxonomic rank.

The alpha diversity indices (i.e., ACE, Chao1, Shannon, and Simpson) of the samples were evaluated using QIIME2 (Bolyen et al., 2019). Beta diversity was analyzed using QIIME to compare the degree of similarity in species diversity across different samples. Principal coordinate analysis (PCoA) was performed on gut microbiota changes in forest musk deer using binary_jaccard. Analysis of Similarities (ANOSIM) was performed to determine the differences among the groups using unweighted_UniFrac. LEfSe [Linear Discriminant Analysis (LDA) Effect Size] was performed to figure out the statistics difference biomarker between different groups. The rank-sum test was used to analyze the significant difference of species in two or more groups and correct the *p*-value using FDR. The function of the microbial community were inferred by PICRUSt (Phylogenetic Investigation of Communities by Reconstruction of Unobserved States) (Douglas et al., 2019) analysis based on 16S rDNA sequencing results.

Alpha diversity indexes are presented as the means ± SD. Statistical analyses were performed with SPSS 20.0. The statistical comparisons were made with the Student’s *t*-test. The level of significance (*P*) was set at 0.05.

## Results

### Analysis of 16S rRNA sequencing results

A total of 1,881,135 pairs of raw reads were obtained from 24 samples. After quality control and splicing, 1,875,191 clean reads were produced—each sample produced at least 58,501 clean reads, with an average of 78,133 clean reads. The statistics of the filtered sequencing data of each sample are shown in Supplementary Table 2 and the effective sequence-length distribution in all samples are shown in Supplementary Figure 1.

**FIGURE 1:**
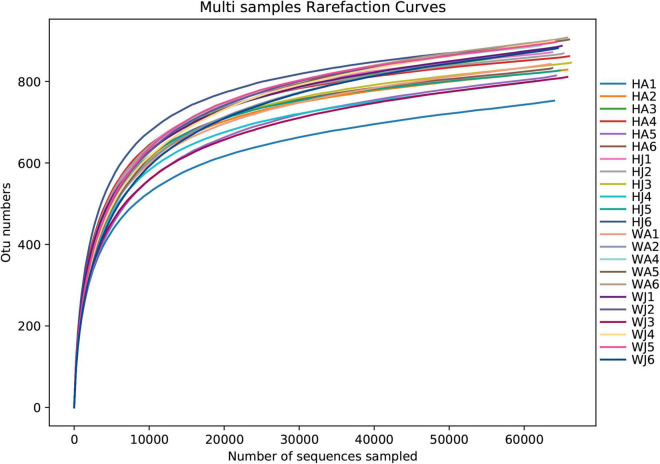
Rarefaction curve. The x-coordinate is the number of sequences sampled and the y-coordinate is the number of observed OTUs. Each curve in the graph represents a sample, which is labeled with a different color. The number of OTUs increases with the sequencing depth. When the curve becomes stable, the number of detected OTUs does not increase with the expansion of extracted data, indicating a time when the amount of sequencing data is reasonable.

Rarefaction curves were used to verify whether the sequencing data was sufficient to reflect the species diversity and indirectly reflect the species abundance of the samples. The final curve became stable, signifying that the sample sequences were sufficient for data analysis (Figure 1). The detected bacteria were classified into 19 phyla, 28 classes, 53 orders, 86 families, 196 genera, and 208 species. The number of each species at different levels are shown in Supplementary Table 3, and the total number of OTUs covered by each sample in their subordinate levels are shown in Supplementary Table 4.

### Bacterial composition and relative abundance

**FIGURE 2:**
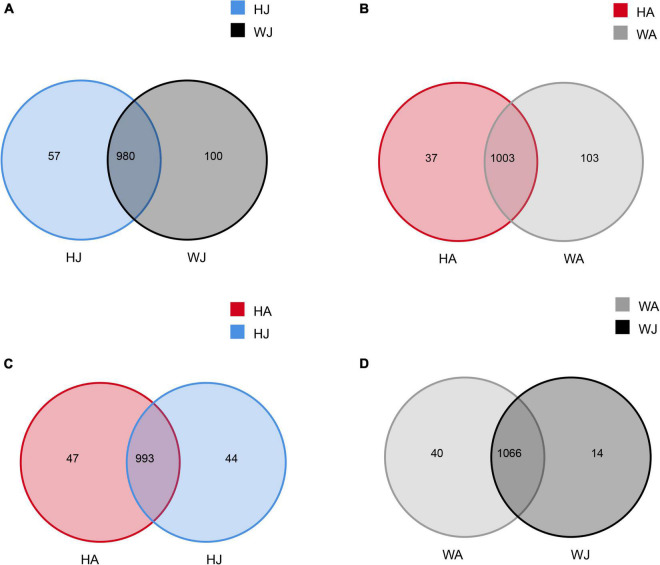
Venn diagram. The Venn diagrams show the numbers of OTUs (97% sequence identity) that were shared or not shared by adult and juvenile forest musk deer in the Weinan and Huailai areas. **(A)** The number of OTUs shared by HJ and WJ. **(B)** The number of OTUs shared by HA and WA. **(C)** The number of OTUs shared by HJ and HA. **(D)** The number of OTUs shared by WJ and WA.

Venn diagrams were used to confirm the core intestinal microbiota of the forest musk deer in the Weinan and Huailai areas. The bacterial populations common to the individuals in each group were considered the core microbiota. As shown in Figure 2, the number of OTUs shared by forest musk deer was 980 in the WJ and HJ groups (Figure 2A), 1,003 in the WA and HA groups (Figure 2B), 993 in the HJ and HA groups (Figure 2C), and 1,066 in the WJ and WA groups (Figure 2D).

**FIGURE 3:**
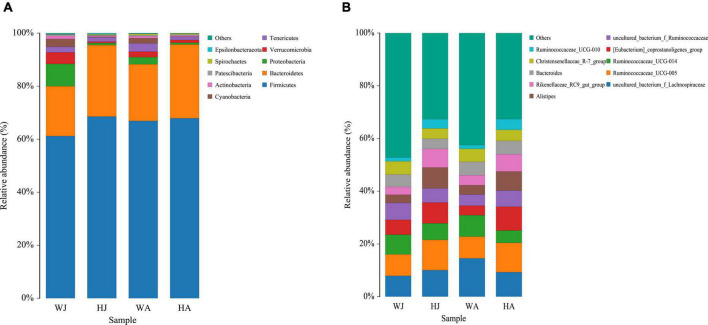
Fecal microbial composition of WJ, HJ, WA, and HA at the phylum **(A)** and genus **(B)** level. Each bar represents the average relative abundance in each group.

The top 10 phyla and 10 genera based on the relative abundance of fecal bacteria in the WJ, HJ, WA, and HA samples are displayed in Figure 3. At phylum level, Firmicutes (61.184%) was the predominant phylum, followed by Bacteroidetes (18.801%) and Proteobacteria (8.401%) in the WJ group. Firmicutes (68.554%) and Bacteroidetes (26.927%) were the dominant phyla, followed by Tenericutes (1.667%) in the HJ group. Firmicutes (66.894%) and Bacteroidetes (21.379%) were the dominant phyla, followed by Tenericutes (3.094%) in the WA group. Firmicutes (67.891%) and Bacteroidetes (27.809%) were the dominant phyla, followed by Tenericutes (1.126%) in the HA group (Figure 3A). At genus level, *Ruminococcaceae_UCG-005, Ruminococcaceae_UCG-014*, and *Christensenellaceae_R-7_group*, all belonging to Firmicutes, were the prevalent genera in the WJ group. *Ruminococcaceae_UCG-005*, *Alistipes*, and the *[Eubacterium]_coprostanoligenes_group* were the most common genera in the HJ group. *Ruminococcaceae_UCG-005*, *Ruminococcaceae_UCG-014*, and *Bacteroides* were the most common genera in the WA group. *Ruminococcaceae_UCG-005, [Eubacterium]_coprostanoligenes_group*, and *Alistipes* were the most common genera in the HA group (Figure 3B).

**FIGURE 4:**
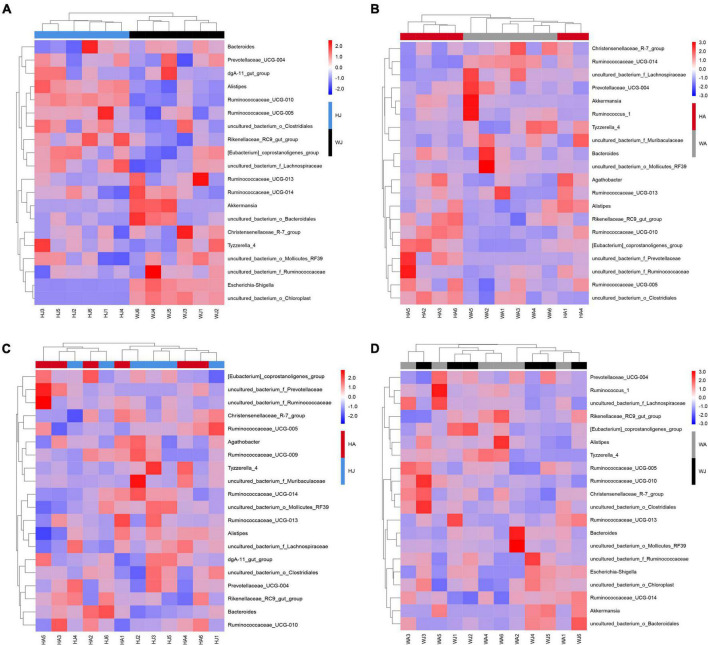
Heatmap analysis. **(A–D)** The 20 most abundant genera between the HJ and WJ, and HA and WA, and HA and HJ, and WA and WJ groups. The corresponding values of the heatmap are the *Z* values obtained by normalizing the relative abundance of genera on each row. The color gradient from blue to red indicates a low to high relative abundance. The vertical clustering indicates the similarity in the richness of different genera among different samples. The closer the distance between two genera, the shorter the branch length, indicating greater similarity in richness between the two genera. Horizontal clustering indicates the similarity of genera richness in different samples. Similarly, the closer the distance between two samples, the shorter the branch length, indicating greater similarity in richness of genera between the two samples.

The heatmap for the clustering of relative abundance of species at the genus level is shown in Figure 4. We selected the top 20 taxa after the normalization (logarithm) of the OTU data. Then, draw the figure based on R heatmap. Each color block in the heatmap represents the abundance of one genus of one sample, with samples arranged horizontally and species arranged vertically. The heatmap clustering shows that there is a separate cluster of the juvenile animals from the HJ and WJ (Figure 4A), whereas there is no separation in the three other comparisons (HA and WA, and HA and HJ, and WA and WJ) (Figures 4B–D).

### Diversity analysis of microbial communities in musk deer in different groups

#### Alpha diversity analysis

**FIGURE 5:**
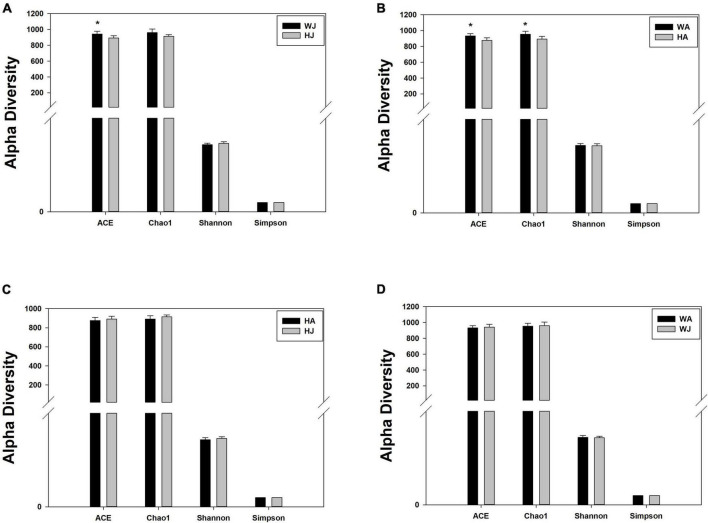
Alpha diversity indices. **(A–D)** Comparison of alpha diversity indices of gut microbiota between the HJ and WJ, and HA and WA, and HA and HJ, and WA and WJ groups. ACE: an index used to estimate the number of OTUs in a community. It is one of the most commonly used indices for estimating species in ecology. Chao: an index that uses the Chao1 algorithm to estimate the number of OTUs included in a sample. Chao is commonly used in ecology to assess the total number of species. Shannon: an index often used to reflect alpha diversity and estimate microbial diversity in a sample. Simpson: a diversity index commonly used in ecology to quantitatively describe the biodiversity of a geographical area. *represents *P* < 0.05.

We calculated the alpha diversity (ACE, Chao1, Shannon, and Simpson) for the gut microbiota in adult and juvenile forest musk deer in the Weinan and Huailai areas (Figure 5). There was a significant difference in the ACE indices between the WJ and HJ group and a significant difference in the ACE and Chao1 indices between the WA and HA group (*P* < 0.05) (Figures 5A,B), but no significant difference was found between the HA and HJ, or WA and WJ groups (*P* > 0.05) (Figures 5C,D).

#### Beta diversity analysis

**FIGURE 6:**
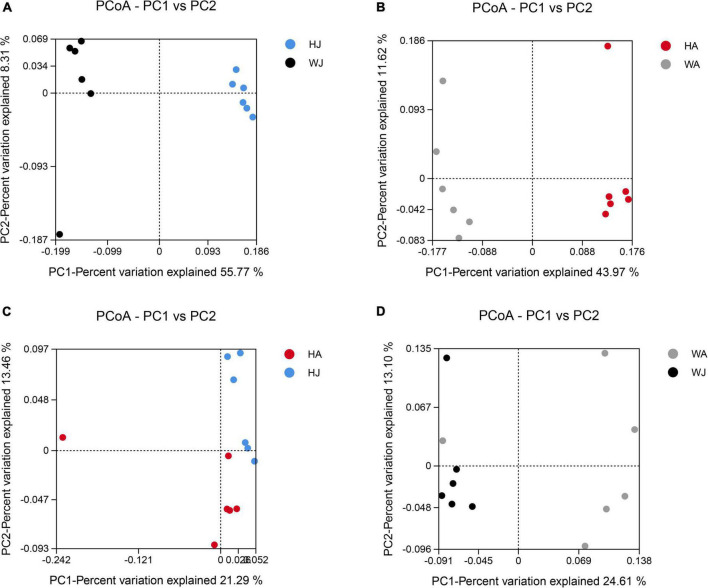
Principal coordinate analysis (PCoA) plot. **(A–D)** Sample differences between the HJ and WJ, and HA and WA, and HA and HJ, and WA and WJ groups. PCoA plot. PC1 vs. PC2 is the PCoA plot obtained from the first and second main coordinates; the *x*-axis and *y*-axis represent the first and second main coordinates, respectively. The percentage of the main coordinates represent the relative contribution of this coordinate to sample differences, which is a measure of the amount of original information extracted by this main coordinate. The distances between the sample points represent the similarity of microbiota in the samples. A closer distance represents higher similarity and samples that cluster together are composed of similar microbiota.

The PC1 vs. PC2 in Figure 6 shows that samples closer together in the graph exhibit greater similarity. The distance between the dots with two colors shows the similarity in the bacterial community structure. The PCoA plot shows the dissimilarity of the microbial communities and reveals distinctly different structures between the HJ and WJ, and HA and WA groups (Figures 6A,B). In the same area, the adults and juveniles indicating a similar structure between the HJ and HA group but a different structure between the WJ and WA group (Figures 6C,D).

**FIGURE 7:**
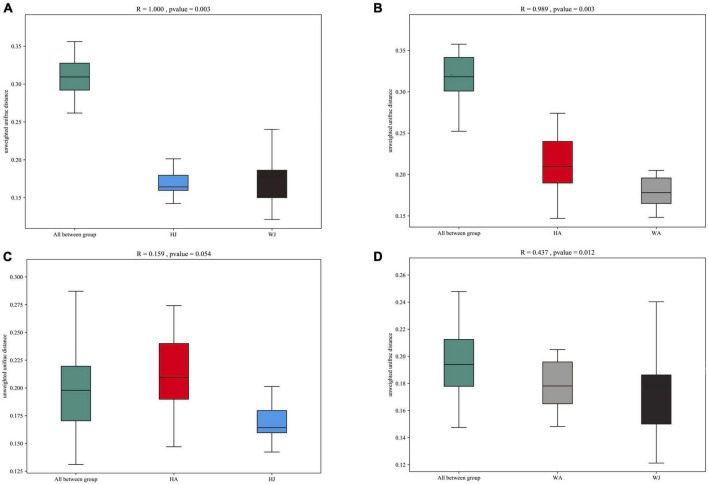
ANOSIM analysis. **(A)** Beta distance of HJ and WJ. **(B)** Beta distance of HA and WA. **(C)** Beta distance of HA and HJ. **(D)** Beta distance of WA and WJ. The *x*-axis represents the grouping and the *y*-axis represents the distance calculated by Unweighted_unifrac. The data in the box is the distance of Inter-group and Intra-group, respectively. The range of R is (−1, 1). *R* > 0 indicates that the difference between groups is greater than that within groups, and *R* < 0 indicates that the difference between groups is smaller than that within groups. *P*-value: *P* < 0.05 indicates high reliability of the test.

Additionally, we used analysis of similarities (ANOSIM) to further test the differences among groups. The inter- and intra-group beta distances are shown in a box plot (Figure 7), with the results showing highly significant differences in bacterial communities between the HJ and WJ (*R* = 1.000, *P* = 0.003 < 0.01), and HA and WA groups (*R* = 0.989, *P* = 0.003 < 0.01) (Figures 7A,B). In the same area, the results show no significant differences in bacterial communities between the HA and HJ groups (*R* = 0.159, *P* = 0.054 > 0.05) but a significant difference in bacterial communities between the WA and WJ group (*R* = 0.437, *P* = 0.012 < 0.05) (Figures 7C,D). The results of ANOSIM were consistent with the PCoA.

### Analysis of differences in gut microbiota in different groups

The LDA effect size (LEfSe) analyses and the rank-sum test were used to determine the specific species that exhibited significant differences between groups.

**FIGURE 8:**
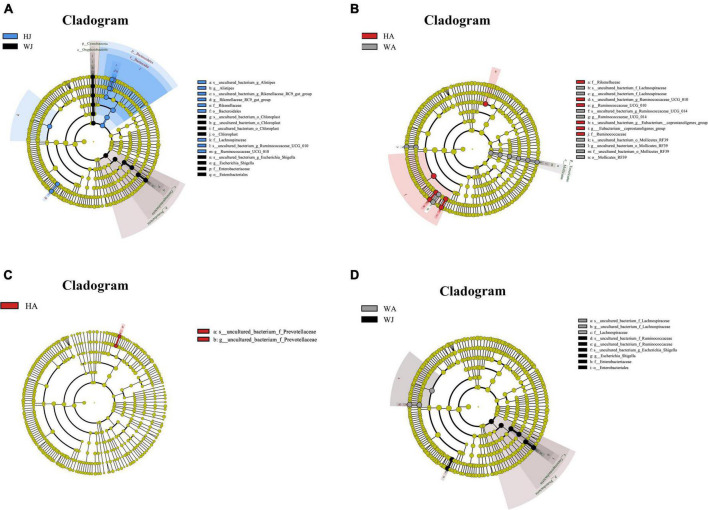
LEfSe (LDA effect size) analysis. **(A–D)** The species that play an important role between the HJ and WJ, and HA and WA, and HA and HJ, and WA and WJ groups. The circle radiated inside-out demonstrated the classification (from phylum to genus). Each small circle at different classification represented a taxa and the diameter of circle is proportional to the relative abundance. The species not with significant differences were colored by yellow and biomarkers were colored by different groups. Red and green dots represent the core bacterial populations in respective group.

Linear discriminant analysis effect size analysis was performed to reveal the significant ranking of abundant modules. The cladogram (Figure 8) displays the microbial taxa with significant differences in adult and juvenile forest musk deer in the Weinan and Huailai areas between different groups. The results showed 12 and 11 taxa with differences in relative abundance in WJ and HJ, respectively (Figure 8A), and 10 and 6 taxa with differences in WA and HA, respectively (Figure 8B). Two taxa in HA (Figure 8C), and eight and three taxa in WJ and WA, respectively (Figure 8D), showed differences in relative abundance.

**FIGURE 9:**
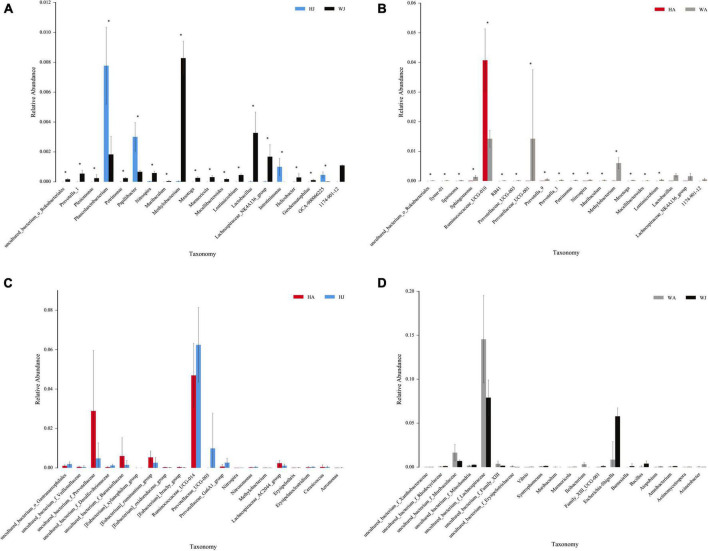
The rank-sum test. **(A–D)** Relative abundance (mean% SD) of 20 major bacterial phyla between the HJ and WJ, and HA and WA, and HA and HJ, and WA and WJ groups. The *x*-axis represents species (relative-abundance differences in the top 20 bacterial communities) and the *y*-axis represents the relative abundance. *represents *P* < 0.05.

The rank-sum test shows the genus-level relative-abundance differences in the top 20 bacterial communities in the forest musk deer samples from the different groups (Figure 9). The relative abundances of *Prevotella-1*, *Plesiomonas*, *Petrimonas*, *Nitrospira*, *Muribaculum*, *Methylobacterium*, *Mesotoga*, *Marmoricola*, *Macellibacteroides*, *Lentimicrobium*, *Lactobacillus*, *Lachnospiraceae_NK4A136_group*, *Helicobacter*, and *Geodermatophilus* in WJ were significantly higher than that in HJ (*P* < 0.05). In contrast, the relative abundance of *Phascolarctobacterium*, *Papillibacter*, *Intestinimonas*, and *GCA-900066225* in WJ were significantly lower than that in HJ (*P* < 0.05). And the relative abundance of *Syner-01*, *Spirosoma*, *Sphingomonas*, *RB41*, *Prevotellaceae_UCG-003*, *Prevotellaceae_UCG-001*, *Prevotella_9*, *Prevotella_1*, *Petrimonas*, *Nitrospira*, *Muribaculum*, *Methylobacterium*, *Mesotoga*, *Macellibacteroides*, and *Lentimicrobium* in WA was significantly higher than that in HA (*P* < 0.05). In contrast, the relative abundance of *Ruminococcaceae_UCG-010* in WA was significantly lower than that in HA (*P* < 0.05) (Figures 9A,B). However, there was no significant difference in the microbial abundance of intestinal microbiota between juvenile and adult musk deer in the same area (Figures 9C,D).

### Phylogenetic investigation of communities by reconstruction of unobserved states function analysis

**FIGURE 10:**
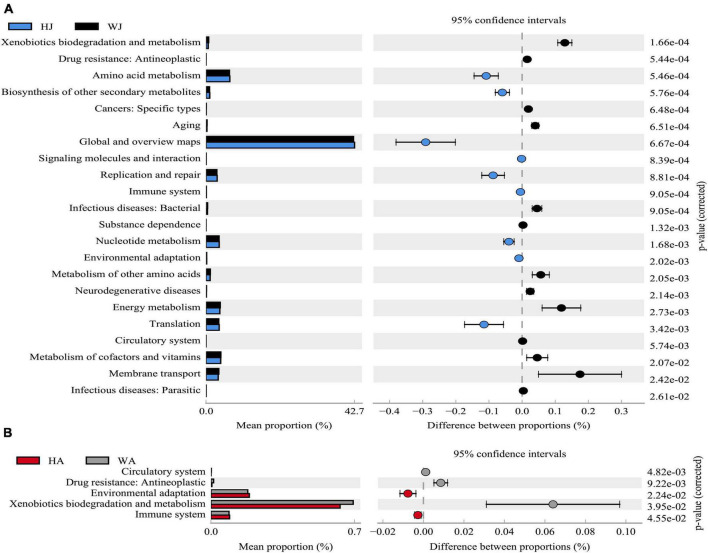
Phylogenetic Investigation of Communities by Reconstruction of Unobserved States analysis. **(A)** The abundance ratio of different functions between HJ and WJ. **(B)** The abundance ratio of different functions between HA and WA. Variance analysis of the KEGG metabolic pathways in the second level. The graphs show the abundance ratio of different functions in two groups of samples. The middle shows the difference between proportions of functional abundance in the 95% confidence interval, and the value at the rightmost is the *P*-value. *P* < 0.05 represents the significant difference.

The results showed that 22 and 5 KEGG level 2 functional genes showed significant differences (*P* < 0.05) between WJ and HJ, and WA and HA, respectively (Figure 10). The differential function genes between WJ and HJ were mainly associated with “Membrane transport,” “Metabolism of cofactors and vitamins,” “Translation,” “Energy metabolism,” “Metabolism of other amino acids,” “Nucleotide metabolism,” “Replication and repair,” “Global and overview maps,” “Amino acid metabolism,” and “Xenobiotics biodegradation and metabolism” (Figure 10A). The differential function genes between WA and HA were mainly associated with “Immune system,” “Xenobiotics biodegradation and metabolism,” and “Environmental adaptation” (Figure 10B).

## Discussion

*Ex situ* conservation is an important technique for protecting rare and endangered wildlife that enables population recovery and rapid expansion. To reduce the risk of maladaptive factors in animals due to *ex situ* conservation, their health status should be continuously monitored. Intestinal health is a vital factor regulating health, and the dynamic equilibrium between intestinal microorganisms and the body affects digestion, absorption of nutrients, metabolism, and immunity (Nicholson et al., 2012; Ottman et al., 2012). Imbalances in intestinal microbiota can cause several serious diseases and severe damage to the body (Wang et al., 2016; Houttu et al., 2020; Wu et al., 2021). The population of forest musk deer that was artificially migrated from Shaanxi to Hebei Province in 2016 increased from approximately 50–260 individuals. However, it was unclear whether the gut microbiota had changed during the *ex situ* conservation process. Consequently, it was necessary to monitor changes in the gut microbiota to assess their health status following *ex situ* conservation and to the best of our knowledge, this is the first research of this kind. In this study, we collected fresh fecal matter from adult and juvenile captive forest musk deer in the Weinan and Huailai areas and performed 16S-rRNA gene Illumina HiSeq sequencing to compare the core gut microbiota between the two populations. The results showed that the intestinal microbiota musk deer from Weinan, Shaanxi exhibited high bacterial species richness. In comparison, the bacterial species richness found in the intestines of musk deer from Huailai, Hebei was significantly lower.

The balance of gut microbiota is influenced by various factors (De Filippo et al., 2010; Scott et al., 2013; Schnorr et al., 2014; Zhao et al., 2019; Li et al., 2020). In our study, the forest musk deer in Hebei Province originated from the Shaanxi Province, and the elapsed time from the arrival of the newly migrated population was not long enough for sufficient generations to pass and for significant genetic changes to occur. Based on this observation, the genetic background of the *ex situ* population can be assumed to be unchanged. Furthermore, during the process of *ex situ* conservation, we considered the impact of the environment on deer population and the potential stress that might be caused by environmental relocation. In this regard, we aimed to select a site that was similar to the wild state of the musk deer and similar to the environment of the original relocation site for breeding. The deer species is warm-blooded with a stable intestinal temperature, hence, the effects of genetics, environmental factors, and temperature on the gut microbiota structure were excluded from consideration. However, after their migration from Shaanxi to Hebei Province, the deer’s diet did not contain added TCM materials because of the limited TCM types and their inaccessibility. In other words, the Shaanxi population had a richer diet while the Hebei population lacked diversity in their diet. Then, we investigated whether this dietary difference may have affected their gut microbiota compositions and indirectly impacted health status.

The relative phylum abundance indicated that Firmicutes and Bacteroidetes were the dominant bacteria in both populations. This finding is consistent with studies on the gut microbiota of ruminant animals, such as the alpine musk deer, sika deer, and Père David’s deer (Guan et al., 2017; Zhang et al., 2018; Sun et al., 2019; Jiang et al., 2021). Firmicutes can process complex carbohydrates, playing an important role in producing short-chain fatty acids from dietary carbohydrates and in maintaining health (Rahat-Rozenbloom et al., 2014; Sheridan et al., 2016). The results demonstrated that Firmicutes were the dominant phylum-level microbiota in each population with no significant density difference between groups, suggesting that it ubiquitously played an important role in energy extraction from non-digested carbohydrates. Bacteroidetes was also an important phylum and was significantly more abundant (*P* < 0.05) in HJ (26.927%) than WJ (18.801%). Previous studies have shown that Bacteroidetes help digest complex carbohydrates, ferment organic matter (Spence et al., 2006), and degrade starch, β-glucan, arabinoxylans, and glycans (Flint et al., 2012). Its abundance reflects the body’s ability to acquire metabolic energy from plant polysaccharides (Wang et al., 2019). Another important phylum observed was Tenericutes, which ranked higher than Proteobacteria. Tenericutes are a unique class of bacteria that lack a cell wall and are typically parasites or commensals of eukaryotic hosts, found in the gut microbiota of both terrestrial (Szekely et al., 2010; Skennerton et al., 2016; Gao et al., 2019) and aquatic animals (Zhang et al., 2014, 2016; Fogarty et al., 2019). Studies have indicated that bacteria of the phyla Tenericutes are associated with illnesses and several crustacean diseases (Ghadersohi and Owens, 1999; Wang et al., 2003; Chen et al., 2011). Hence, more attention should be paid to the higher relative abundance and function of Tenericutes in forest musk deer.

Heatmap clustering showed that significantly different gut microbiota structures formed between the juvenile forest musk deer in the two areas. This was not demonstrated in the adult populations between the areas, nor between the adults and juveniles of the same area. We hypothesized that this phenomenon occurred because the gut microbiota structure was influenced by comprehensive factors. In juveniles, the gut microbiota is likely to be more affected by food intake, and thus the differences in food variety between the two regions led to significant differences in the juvenile deer’s gut microbiota. As the deer mature, genetic effects may have a stronger influence, however, as the forest musk deer in Weinan, Shaanxi, and Huailai, Hebei have the same genetic background, there is no complete separation between the individuals of WA and HA. Further, the identical genetic background, environment, and food of forest musk deer in the same geographical location results in similar gut microbiota. These results and observations also verified the important role of food in shaping the gut microbiota of forest musk deer during *ex situ* conservation.

The results of alpha diversity analysis revealed that the microbiota richness was greater in the forest musk deer of the Weinan area compared with the Huailai area, however, forest musk deer within the same areas did not exhibit significant differences in alpha indices, indicating that those individuals had comparable gut microbiota richness and diversity. Additionally, the comparison of beta diversity showed significant differences in the gut microbiota communities between different areas, however, deer in the same region tended to be similar. The above results again suggest that a reduction in food variety may have affected the gut microbiota of forest musk deer after *ex situ* conservation, resulting in a significant reduction in the richness and a significantly different structure of gut microbiota in the artificially migrated forest musk deer. This is similar to previous observations that found wild animals to exhibit greater gut microbiota richness than those in captivity because animals in the wild can forage freely with access to diverse food types and sources. Furthermore, the abundant food sources could promote higher diversity colonization of gut microbiota (Passos et al., 2018; Chi et al., 2019; Jiang et al., 2020).

The rank-sum test indicated that the richness of non-predominant intestinal microbiota was significantly decreased in the migrated individuals compared to those in the Shaanxi region. Most of these non-predominant intestinal microbiota are regarded as beneficial bacteria that could facilitate digestion and absorption and keep intestinal health such as *Prevotella_1, Prevotellaceae_UGG-003*, *Prevotellaceae_UGG-001*, *Prevotella_1*, *Prevotellaceae_9*, and *Lactobacillus* and *Lachnospiraceae_NK4A136_group*. These beneficial bacteria contribute to the utilization of plant materials by degrading glycoproteins, promoting host growth, and facilitating digestion by degradation of hemicellulose and xylan. They also participate in carbohydrate and protein digestion (Rho et al., 2005; Biddle et al., 2013; Chen et al., 2017; Tian et al., 2021; Wang et al., 2022). These results indicate that the gut microbiota of forest musk deer in the emigration area had a higher richness of beneficial bacteria for gut health, digestion, and absorption compared with those in the migrated forest musk deer. This further infers that lower food variety was the cause of lower gut microbiota diversity.

Phylogenetic investigation of communities by reconstruction of unobserved states analysis demonstrated that the abundance of functional genes for the metabolic pathways of digestion and absorption of gut nutrients, and the degradation of harmful substances significantly decreased in the migrated deer compared to those of the Shaanxi region. Intestinal microbiota are a key component of digestion, breaking down complex carbohydrates, proteins, and fibers (Oliphant and Allen-Vercoe, 2019) and it is closely related to the digestive functions of the host. The results determined that after *ex situ* conservation, reduced food diversity influenced the digestive function of forest musk deer, which in turn further affected health status. In view of this, to maintain the stability of intestinal microbiota function in forest musk deer after *ex situ* conservation, the feeders should pay close attention to the composition and diversity of feeds.

To date, forest musk deer breeding in China has mainly been conducted in two regions: the Qinling mountainous area in southern Shaanxi Province and the western mountainous area in Sichuan Province. These two regions account for 90% of the captive musk deer population in China, of which Shaanxi Province comprises 70%. The original forest musk deer resources in other areas of China have almost become completely extinct and very few of the forest musk deer artificial breeding programs have been performed on a large scale. Therefore, to rapidly restore the population and historical distribution of forest musk deer, promoting their *ex situ* conservation is a potential future trend. Our findings indicate that sites with migrated or captive forest musk deer should focus on the influence of dietary differences, feeding methods, and the supplementation of feed types to improve the deer’s intestinal microbiota richness to ensure intestinal health during *ex situ* breeding. Further, we showed that TCMs might have good potential as a feed supplement in animal production as they can improve the gut microbiota abundance (especially beneficial bacteria) and enhance the gut’s ability to digest and absorb nutrients and degrade toxins. The artificial addition of TCMs ensures that the diet of captive forest musk deer is similar to that of their wild counterparts, which is particularly important during *ex situ* breeding. Hence, investigating the effect of TCMs on the function of the gut microbiota of forest musk deer and developing adequate feed additives should be explored.

## Data availability statement

The datasets presented in this study can be found in online repositories. The names of the repository/repositories and accession number(s) can be found below: https://www.ncbi.nlm.nih.gov/.

## Ethics statement

The animal study was reviewed and approved by the Ethics and Animal Welfare Committee of Beijing Forestry University (Approval No. EAWC_BJFU_20200001).

## Author contributions

YJ: conceptualization, validation, formal analysis, and writing – original draft, review and editing. XH: conceptualization, formal analysis, software, and writing – review and editing. ML and NF: investigation and writing – review and editing. PY, HZ, and CZ: investigation, software, and writing – review and editing. MS: formal analysis and writing – review and editing. ZH and RS: sample collection. SL: conceptualization, methodology, supervision, writing – review and editing, and project administration. DH: conceptualization and project administration. All authors contributed to the article and approved the submitted version.
