# Supplementary figures and images for: Changes in the gut microbiota of forest musk deer (Moschus berezovskii) during ex situ conservation
Source: Front Microbiol. 2022 Sep 8;13:969593. doi: 10.3389/fmicb.2022.969593 (PMC9493438; doi:10.3389/fmicb.2022.969593)

# Length Distribution

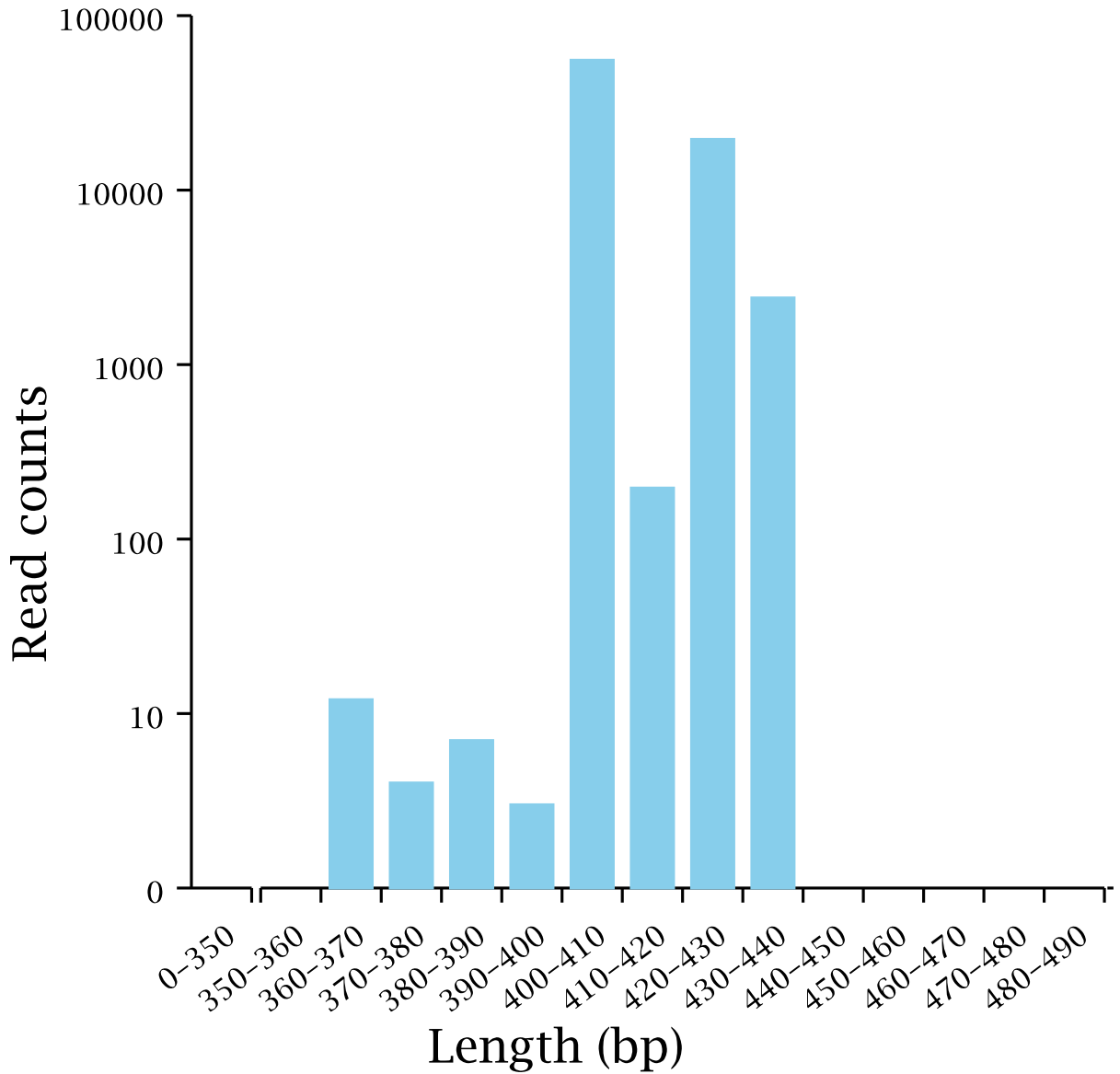

Supplement: Supplementary file 1 [file Data_Sheet_1.ZIP › Supplementary Figure S1/HA1_reads_length/HA1_reads_length.pdf]

# Length Distribution

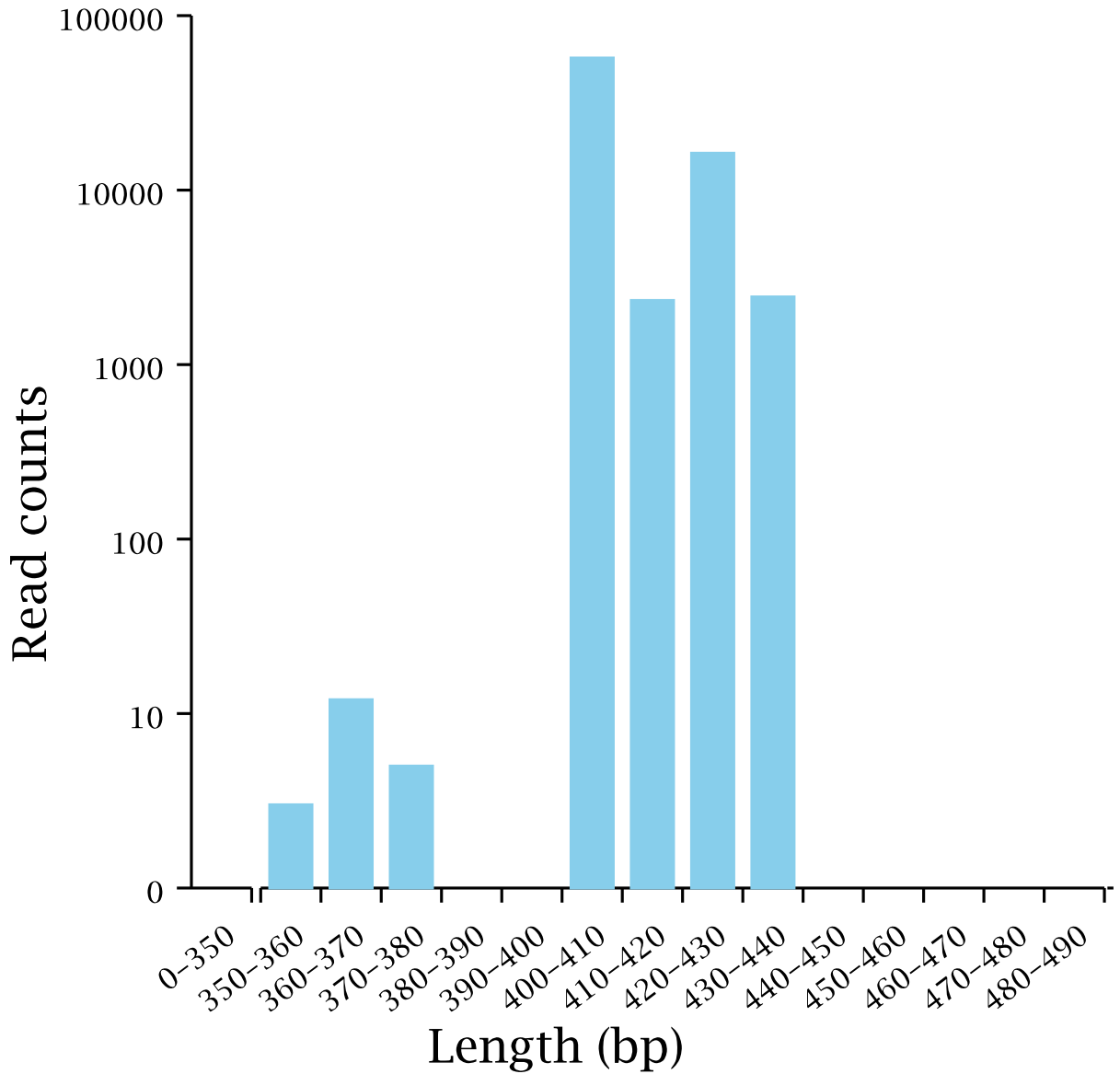

Supplement: Supplementary file 1 [file Data_Sheet_1.ZIP › Supplementary Figure S1/HA2_reads_length/HA2_reads_length.pdf]

# Length Distribution

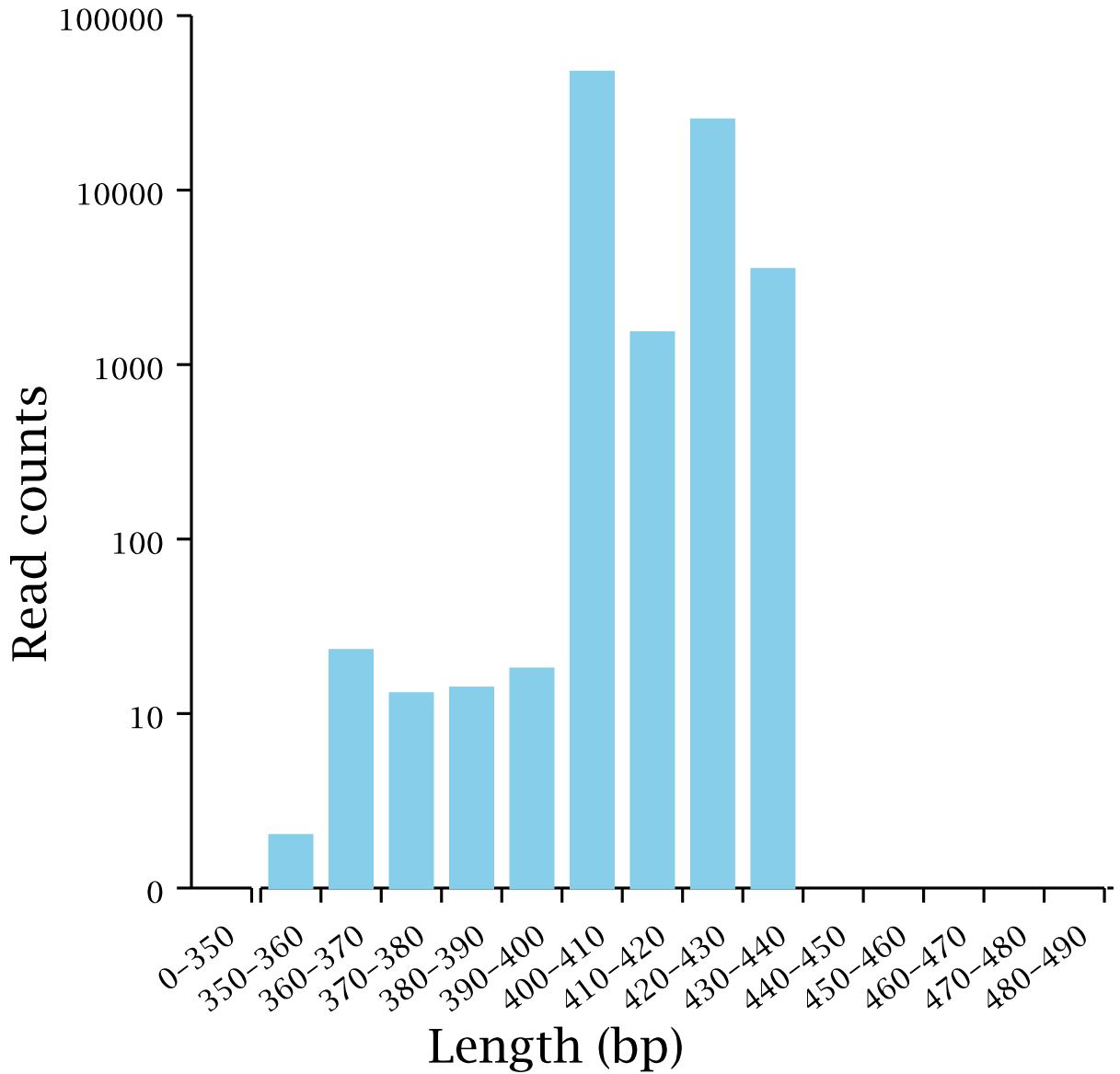

Supplement: Supplementary file 1 [file Data_Sheet_1.ZIP › Supplementary Figure S1/HA3_reads_length/HA3_reads_length.pdf]

# Length Distribution

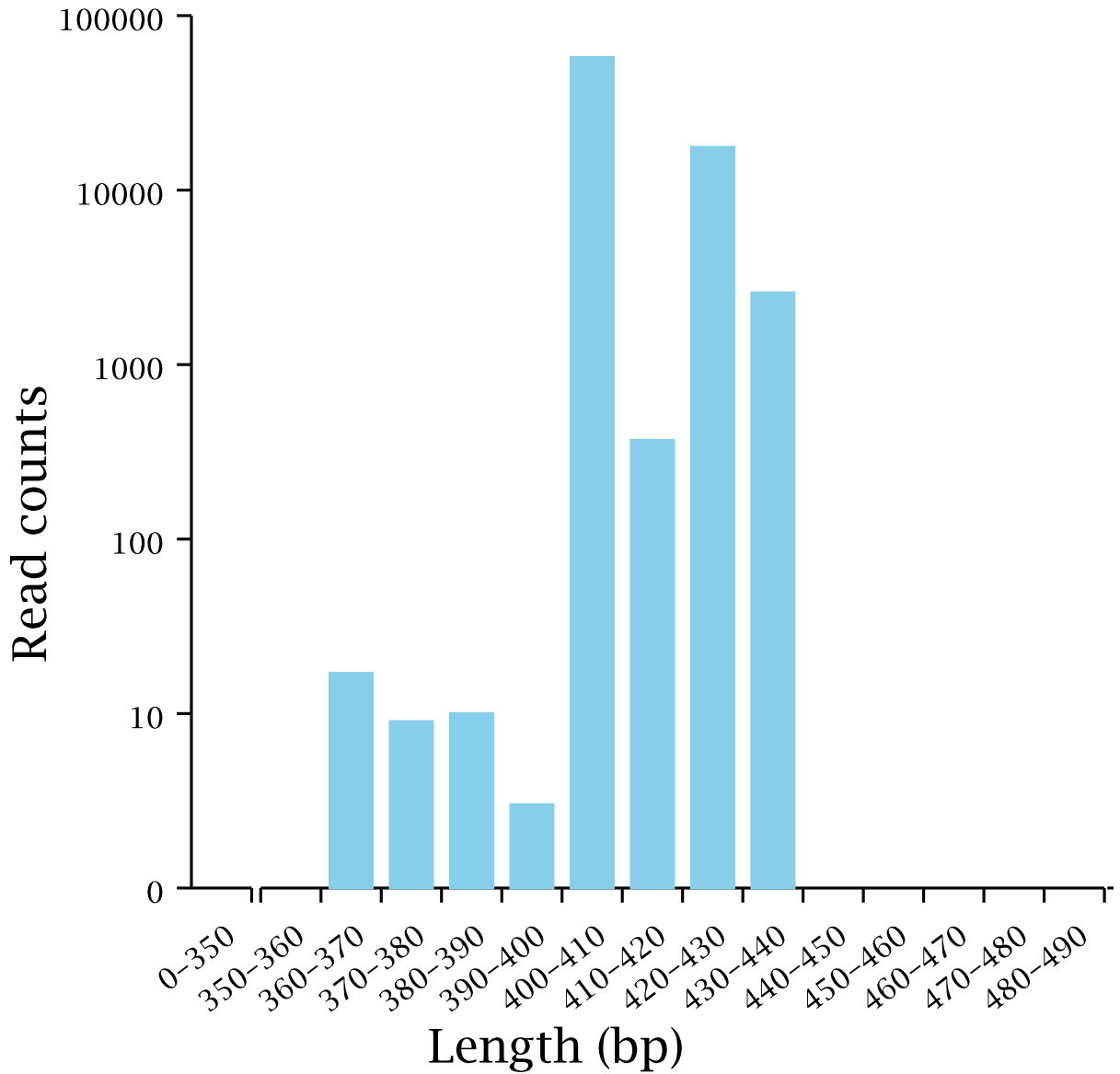

Supplement: Supplementary file 1 [file Data_Sheet_1.ZIP › Supplementary Figure S1/HA4_reads_length/HA4_reads_length.pdf]

# Length Distribution

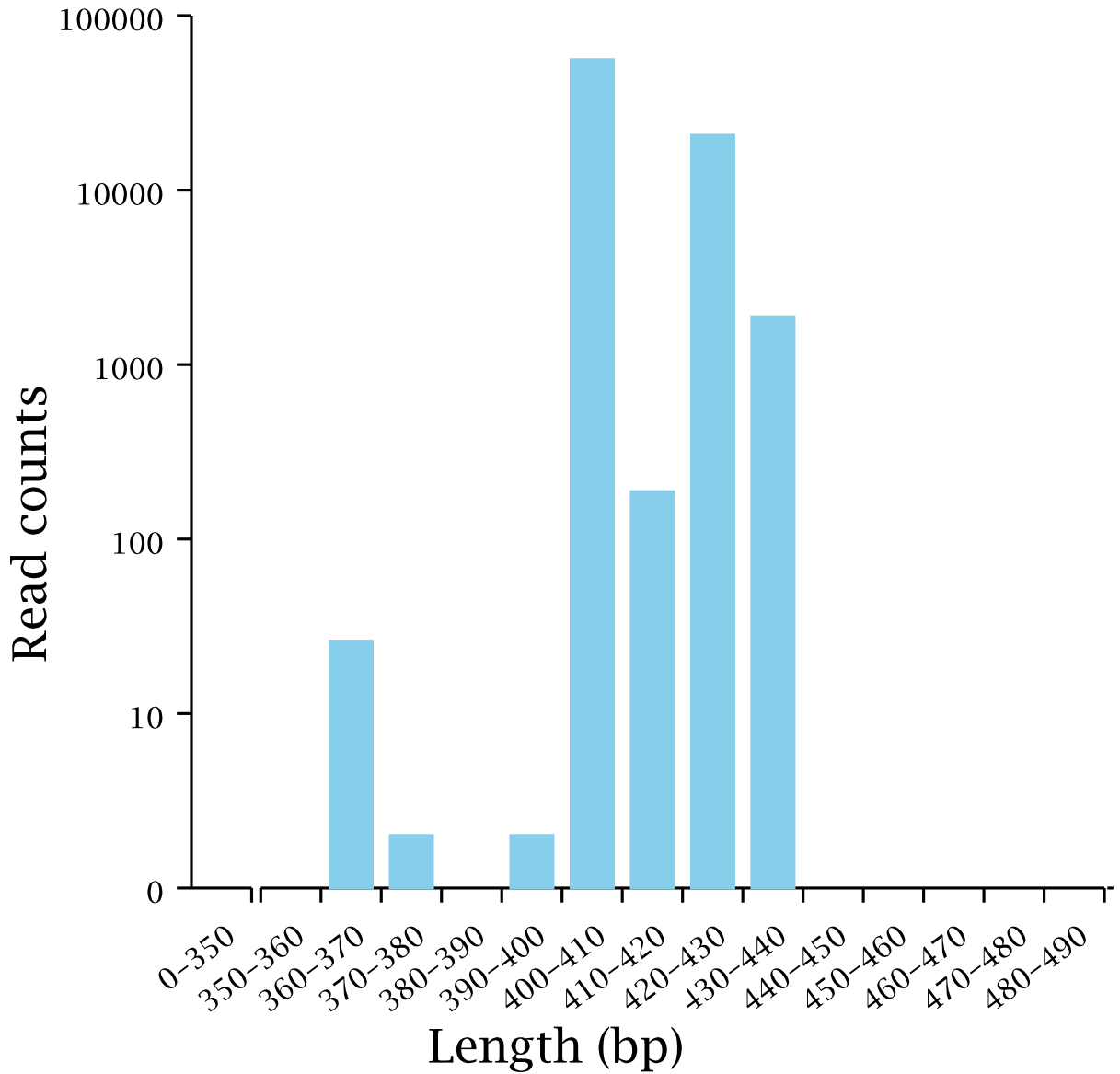

Supplement: Supplementary file 1 [file Data_Sheet_1.ZIP › Supplementary Figure S1/HA5_reads_length/HA5_reads_length.pdf]

# Length Distribution

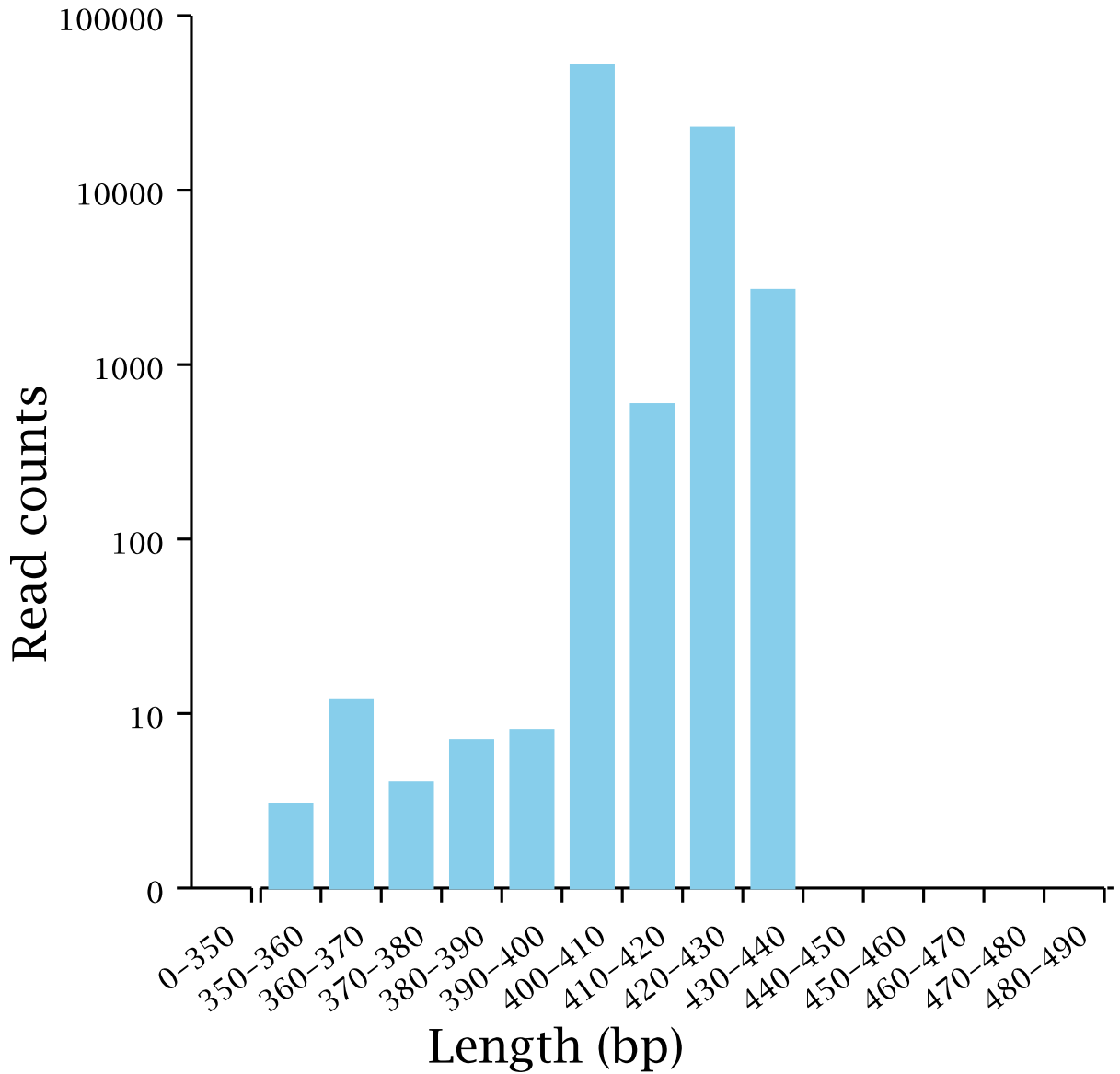

Supplement: Supplementary file 1 [file Data_Sheet_1.ZIP › Supplementary Figure S1/HA6_reads_length/HA6_reads_length.pdf]

# Length Distribution

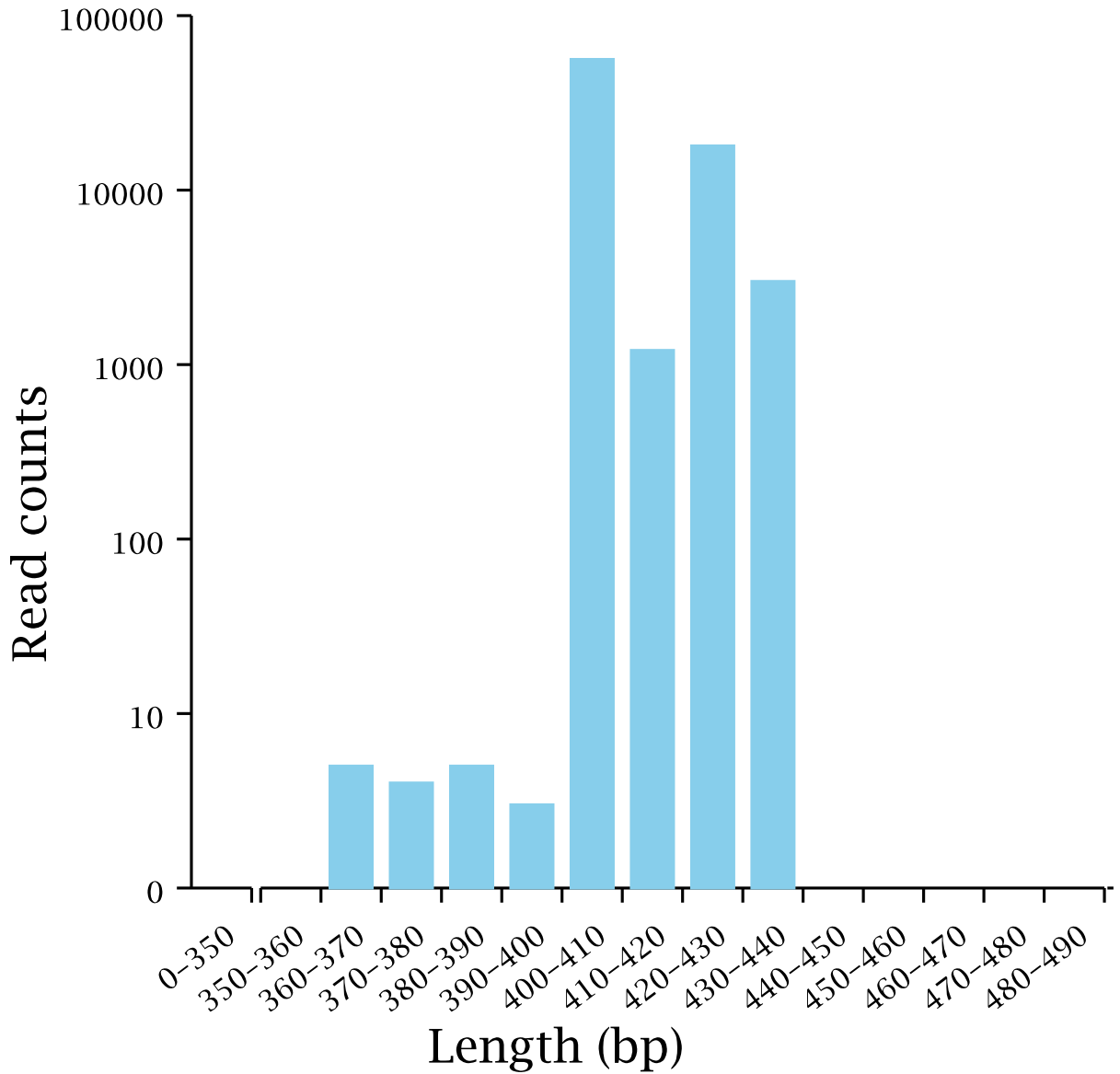

Supplement: Supplementary file 1 [file Data_Sheet_1.ZIP › Supplementary Figure S1/HJ1_reads_length/HJ1_reads_length.pdf]

# Length Distribution

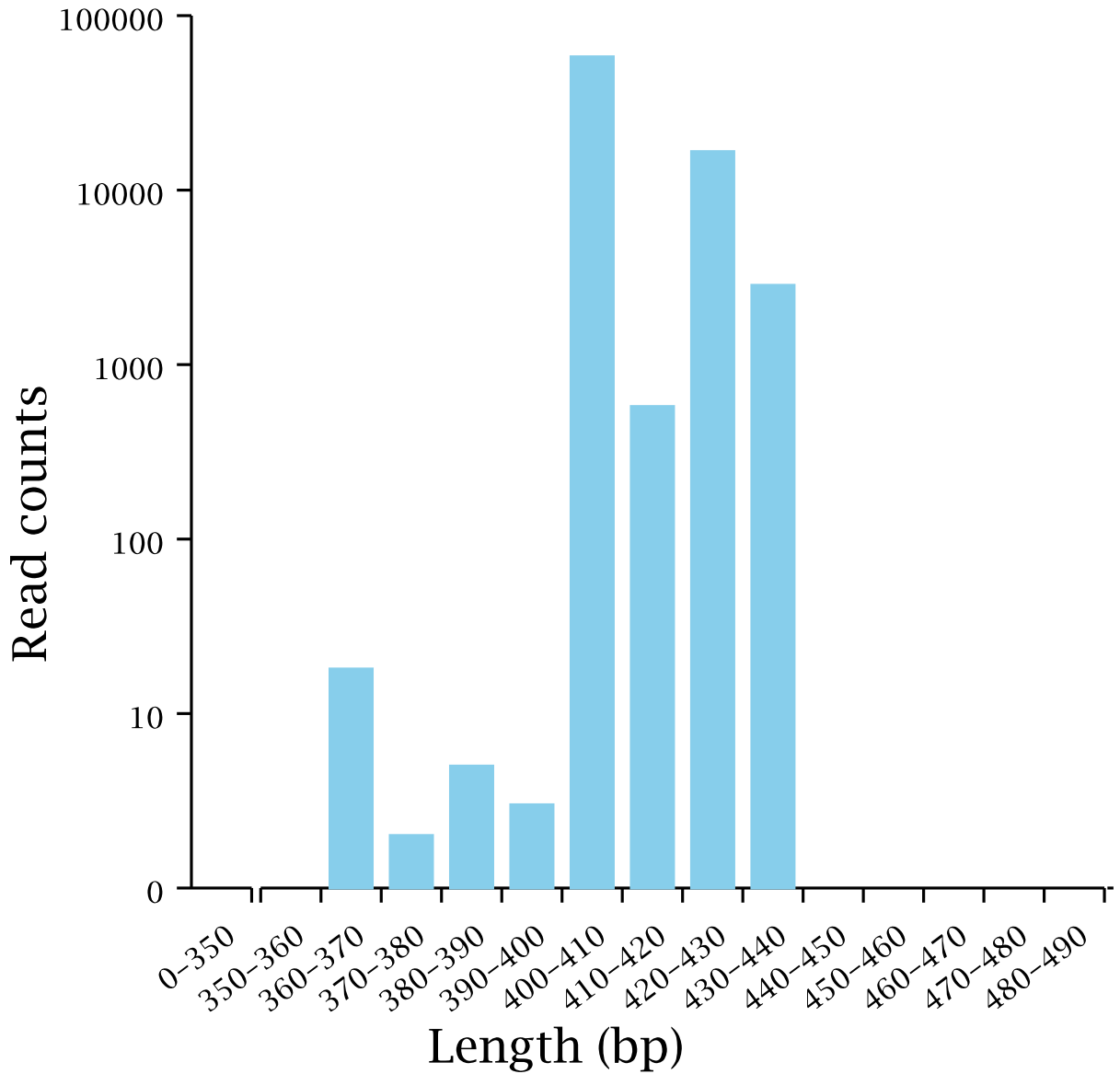

Supplement: Supplementary file 1 [file Data_Sheet_1.ZIP › Supplementary Figure S1/HJ2_reads_length/HJ2_reads_length.pdf]

# Length Distribution

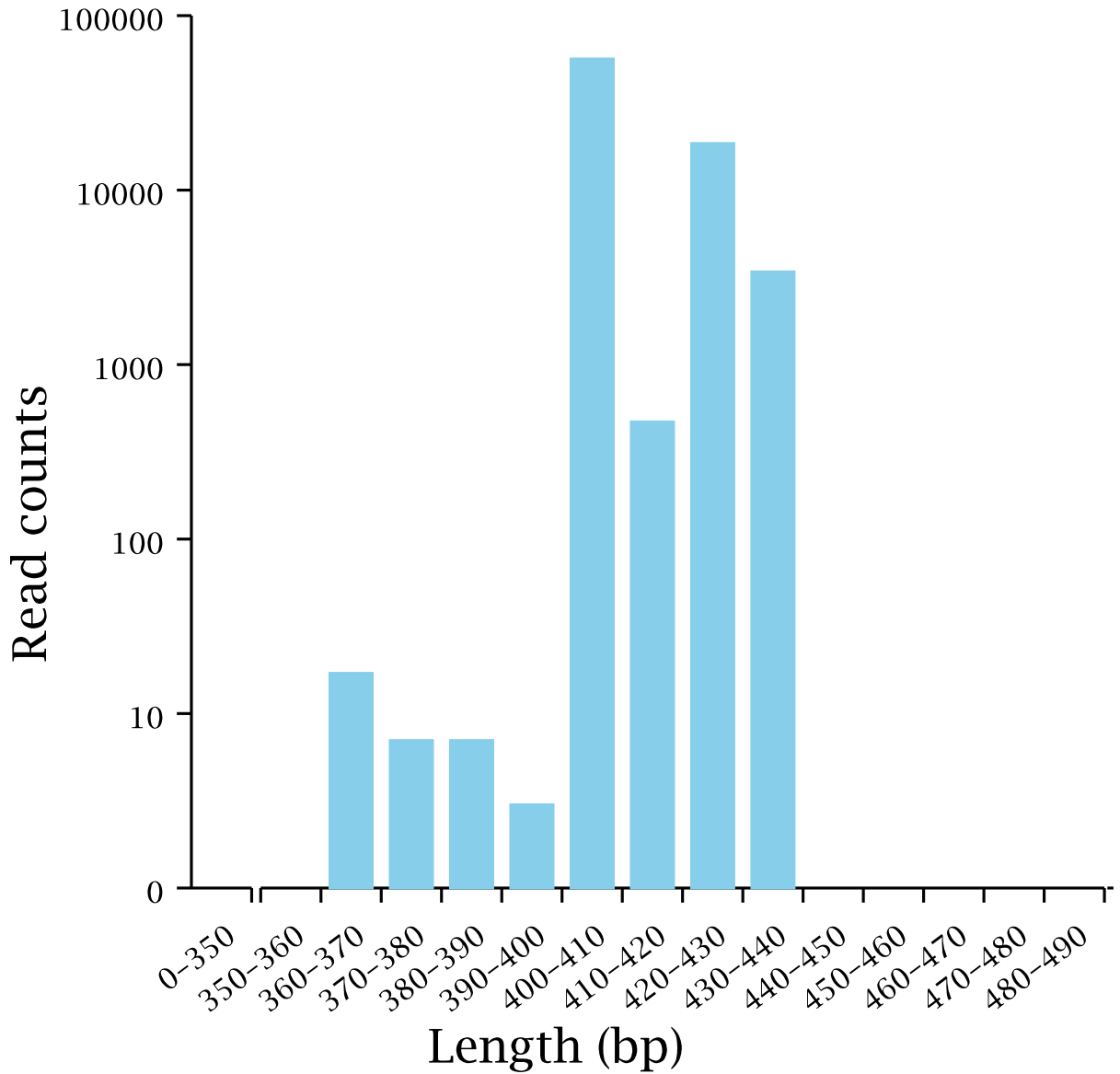

Supplement: Supplementary file 1 [file Data_Sheet_1.ZIP › Supplementary Figure S1/HJ3_reads_length/HJ3_reads_length.pdf]

# Length Distribution

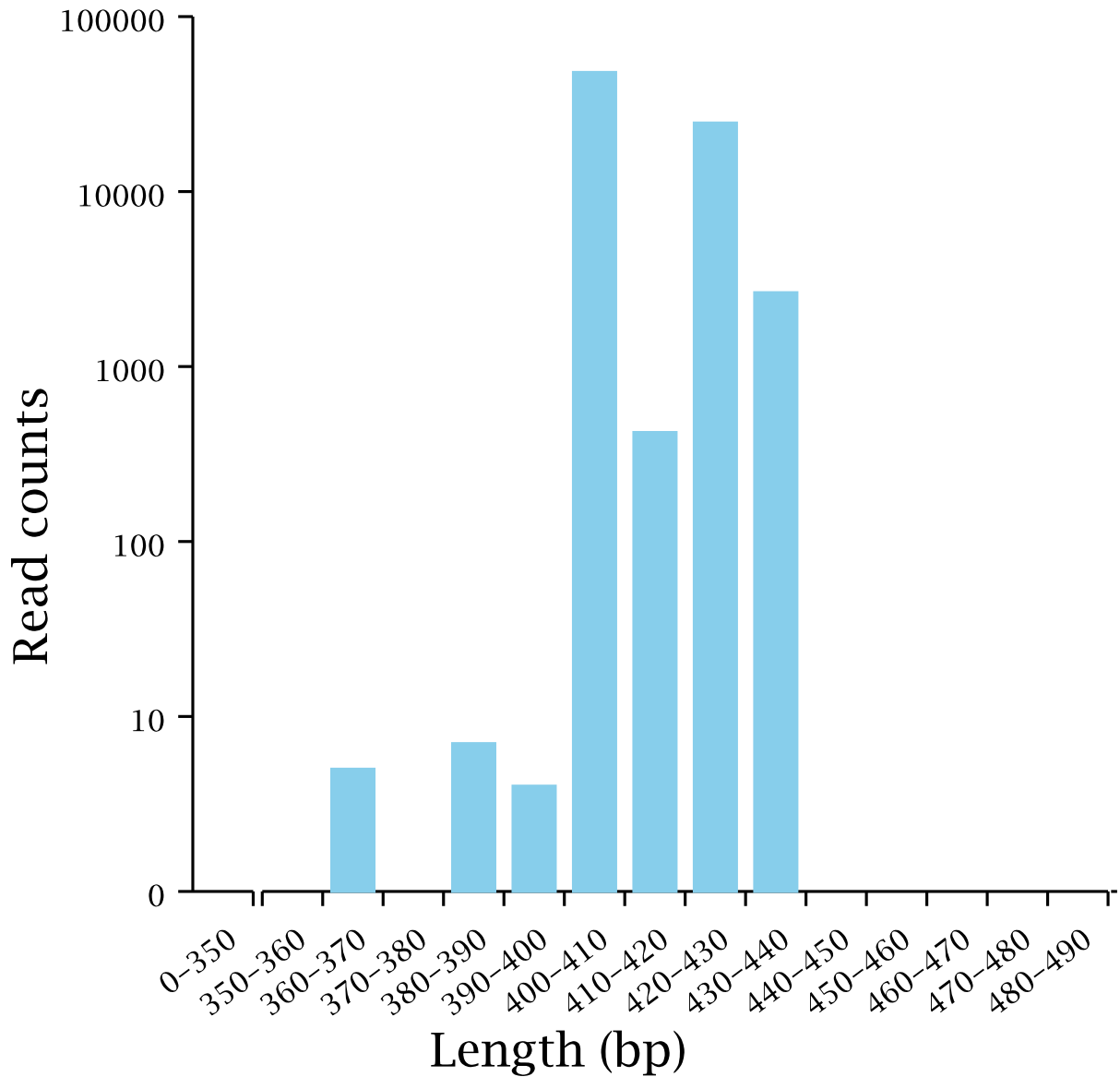

Supplement: Supplementary file 1 [file Data_Sheet_1.ZIP › Supplementary Figure S1/HJ4_reads_length/HJ4_reads_length.pdf]

# Length Distribution

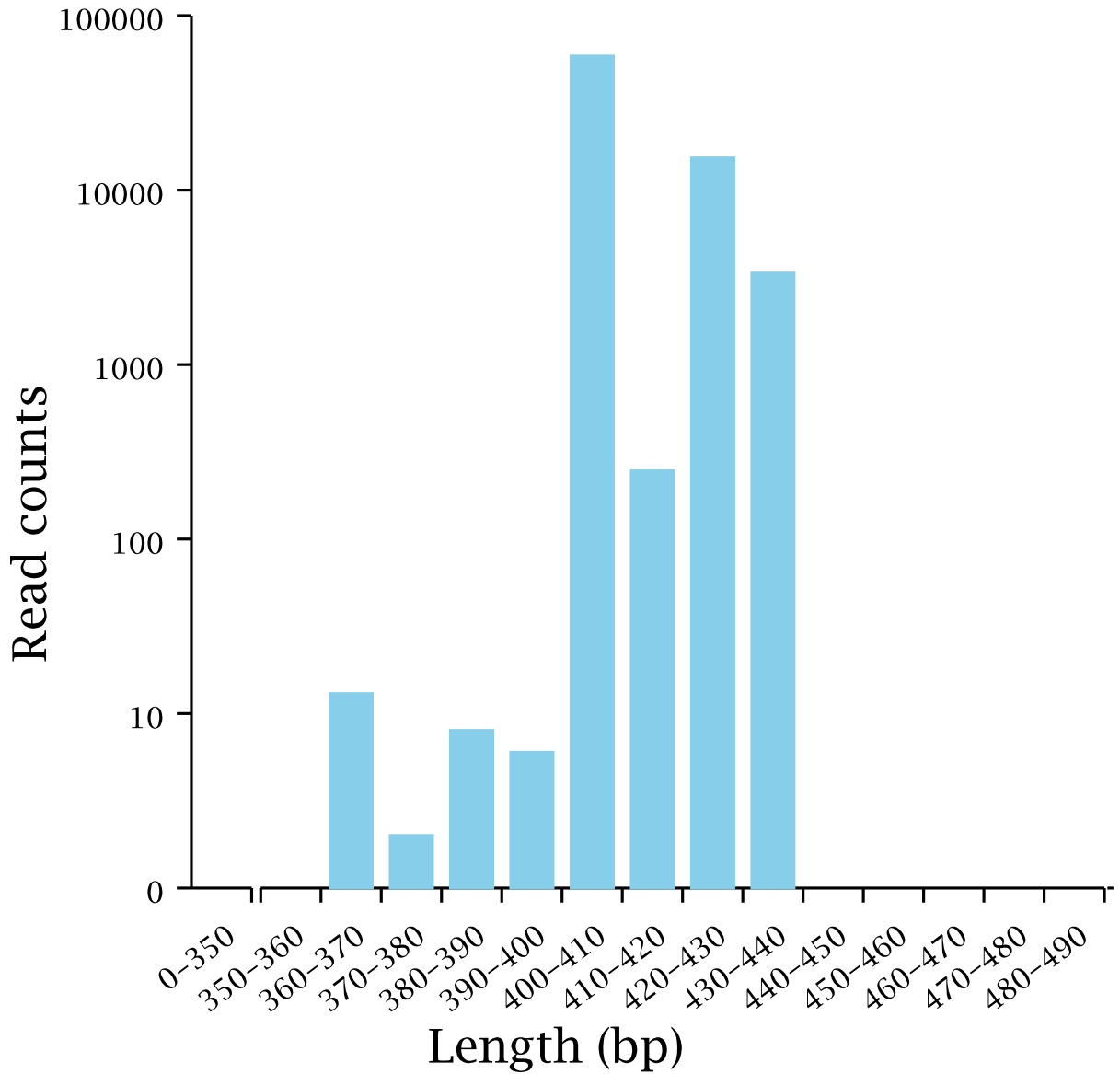

Supplement: Supplementary file 1 [file Data_Sheet_1.ZIP › Supplementary Figure S1/HJ5_reads_length/HJ5_reads_length.pdf]

# Length Distribution

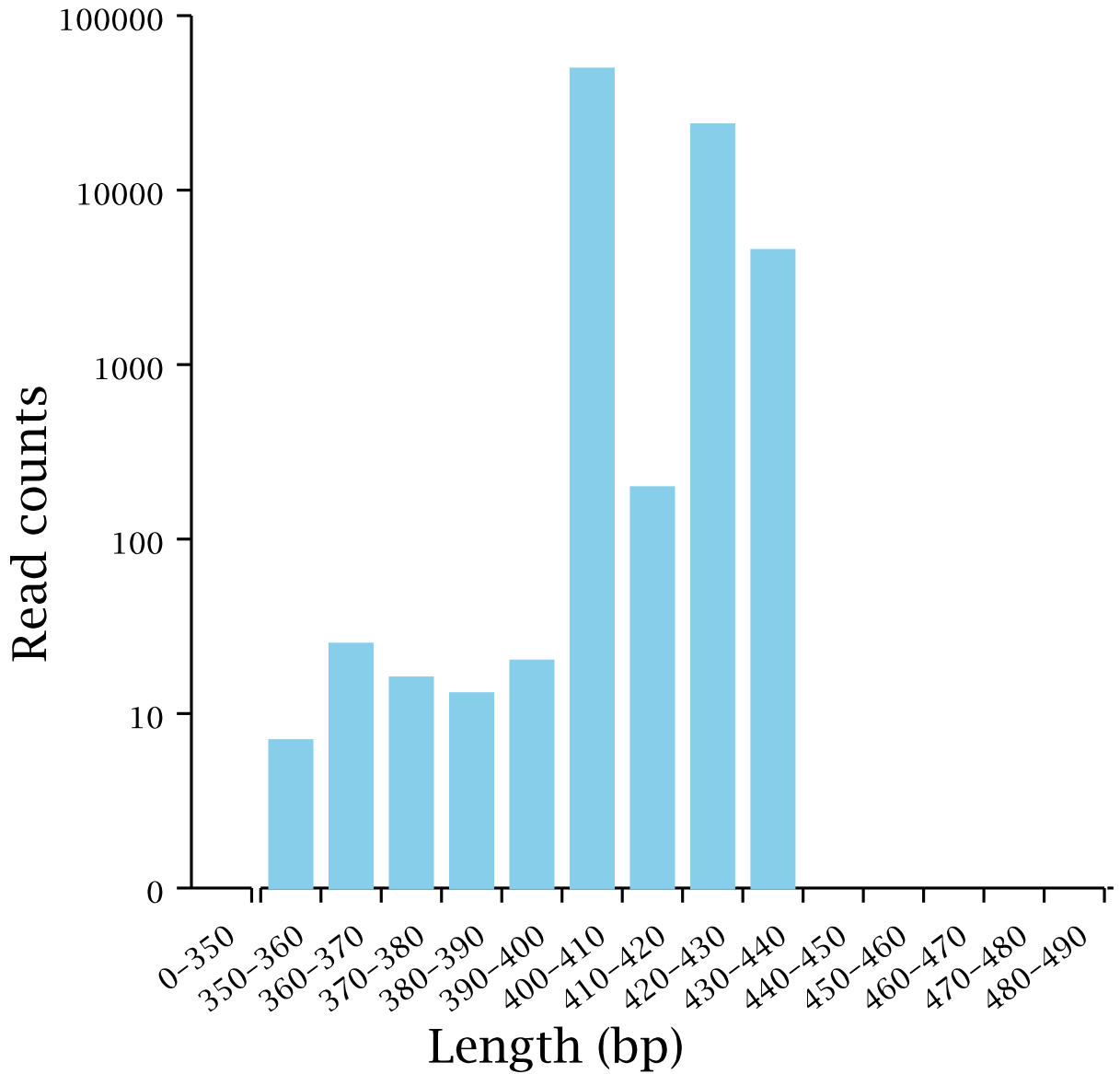

Supplement: Supplementary file 1 [file Data_Sheet_1.ZIP › Supplementary Figure S1/HJ6_reads_length/HJ6_reads_length.pdf]

# Length Distribution

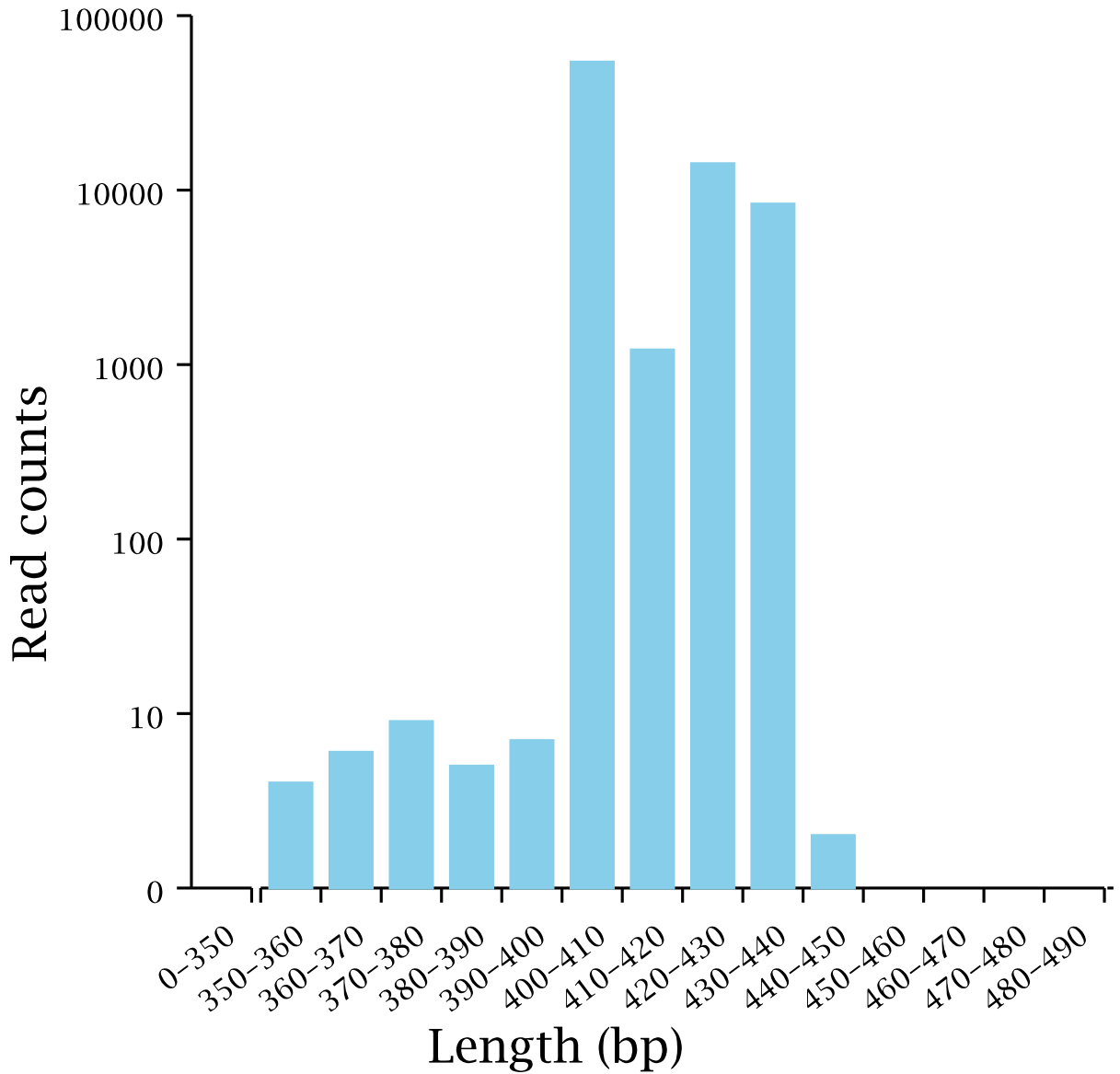

Supplement: Supplementary file 1 [file Data_Sheet_1.ZIP › Supplementary Figure S1/WA1_reads_length/WA1_reads_length.pdf]

# Length Distribution

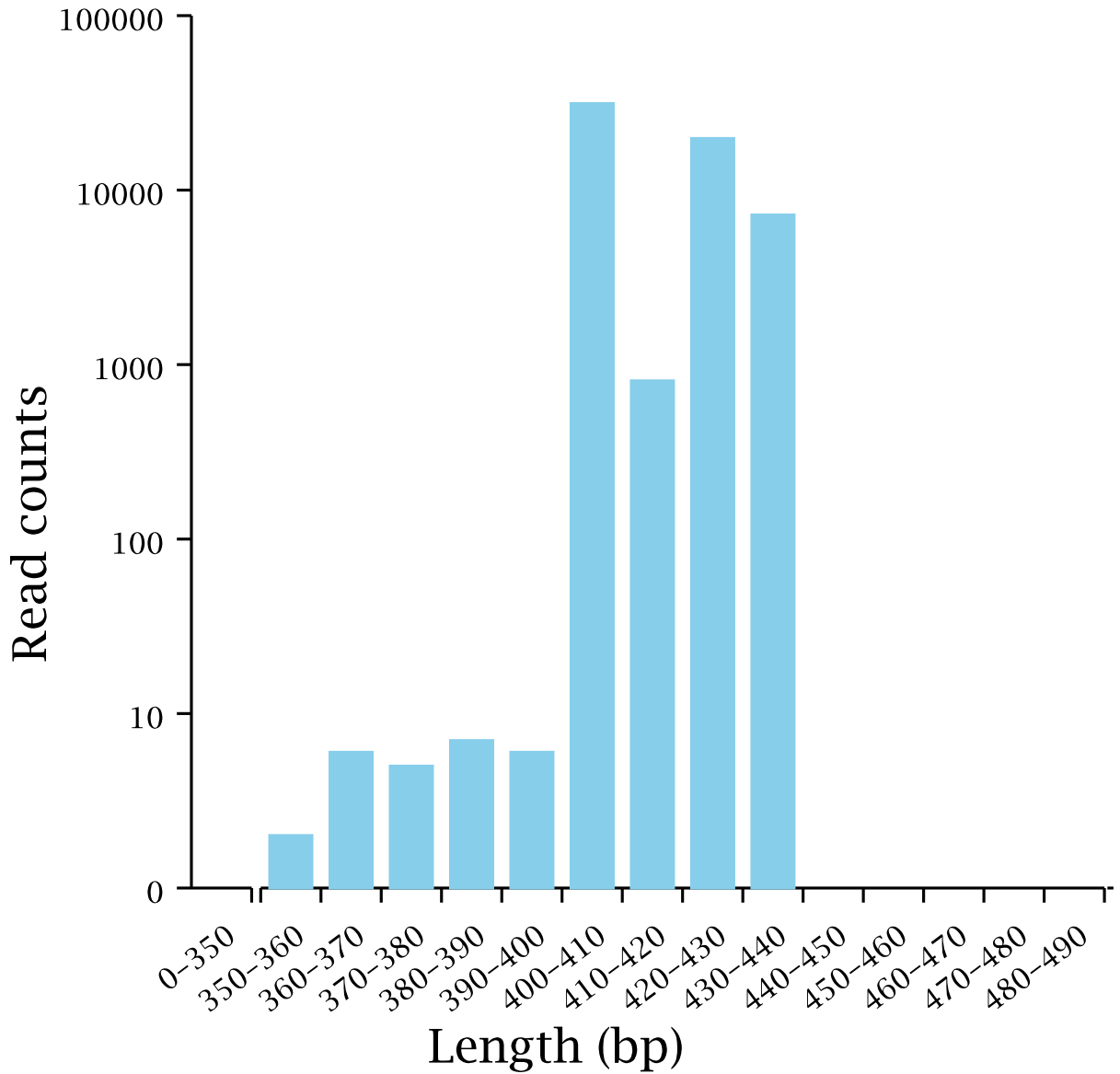

Supplement: Supplementary file 1 [file Data_Sheet_1.ZIP › Supplementary Figure S1/WA2_reads_length/WA2_reads_length.pdf]

# Length Distribution

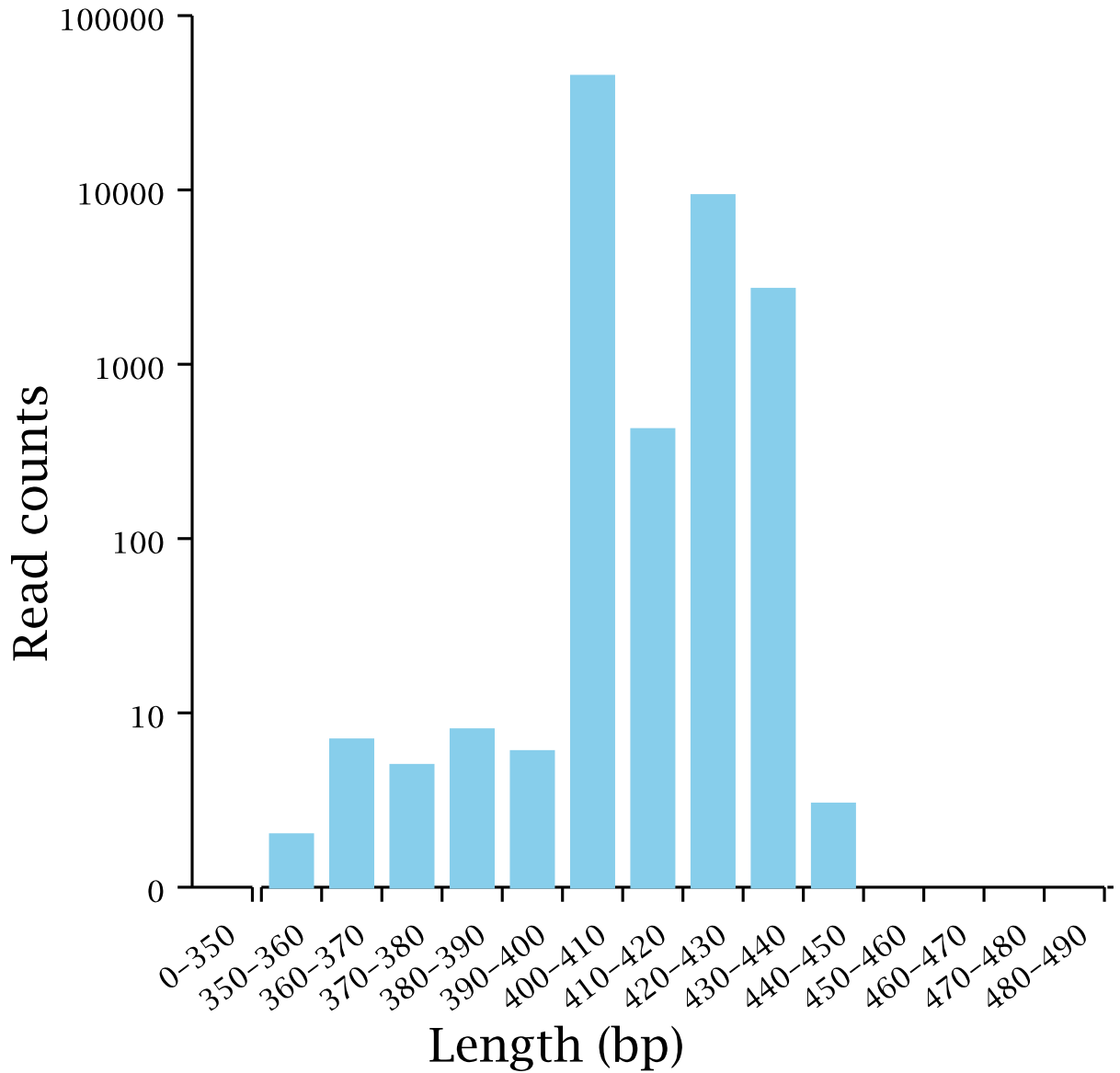

Supplement: Supplementary file 1 [file Data_Sheet_1.ZIP › Supplementary Figure S1/WA3_reads_length/WA3_reads_length.pdf]

# Length Distribution

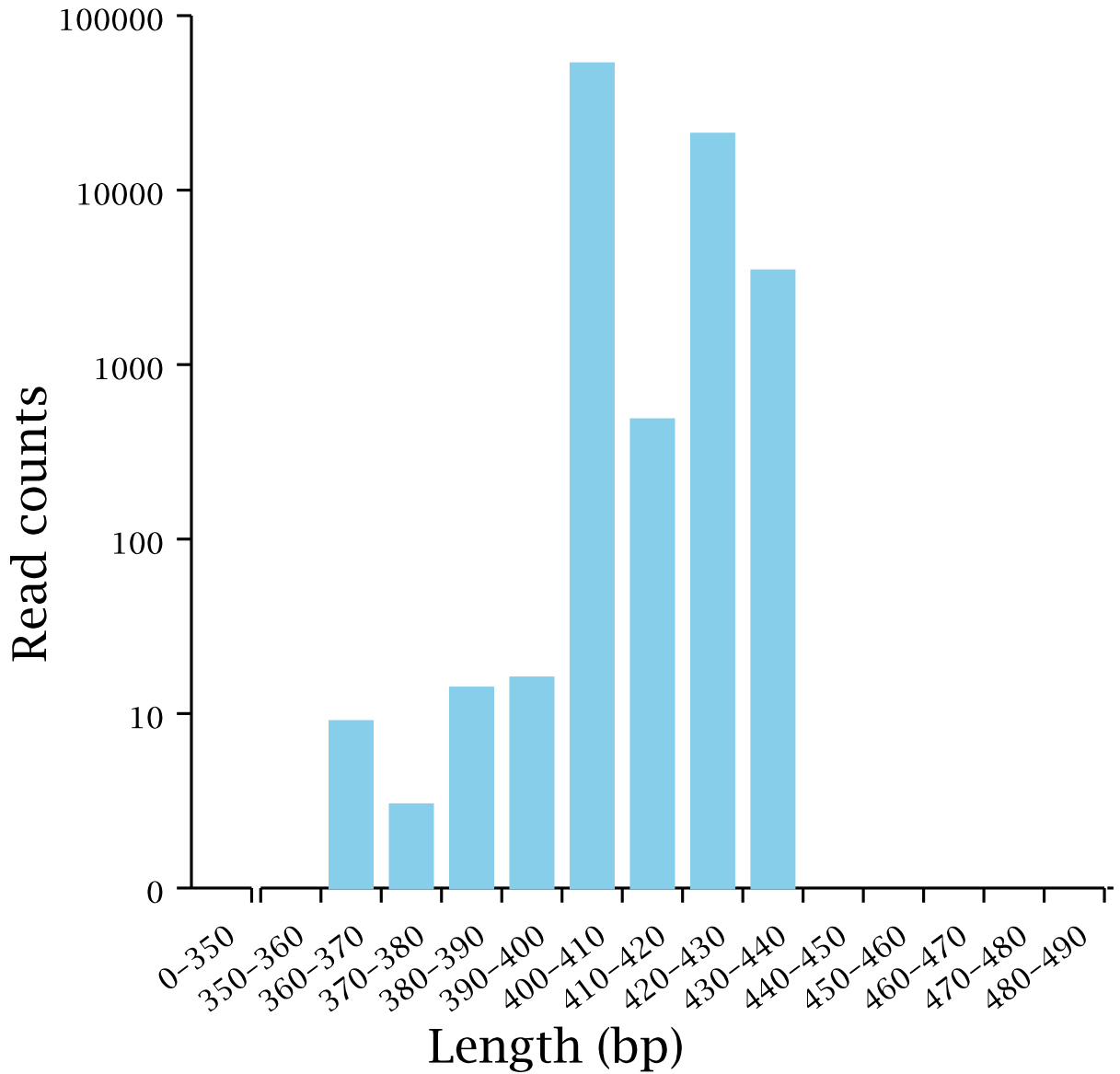

Supplement: Supplementary file 1 [file Data_Sheet_1.ZIP › Supplementary Figure S1/WA4_reads_length/WA4_reads_length.pdf]

# Length Distribution

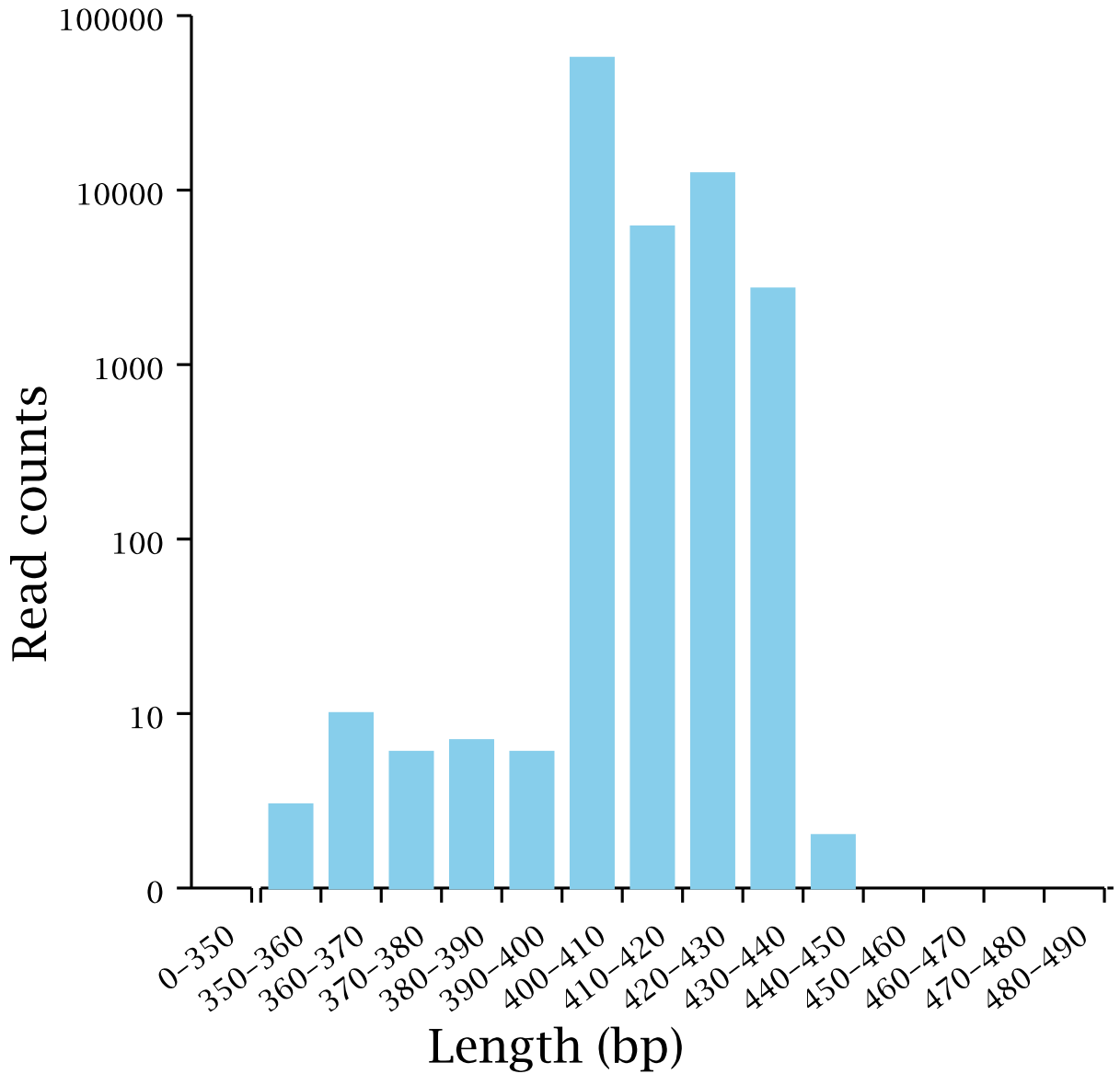

Supplement: Supplementary file 1 [file Data_Sheet_1.ZIP › Supplementary Figure S1/WA5_reads_length/WA5_reads_length.pdf]

# Length Distribution

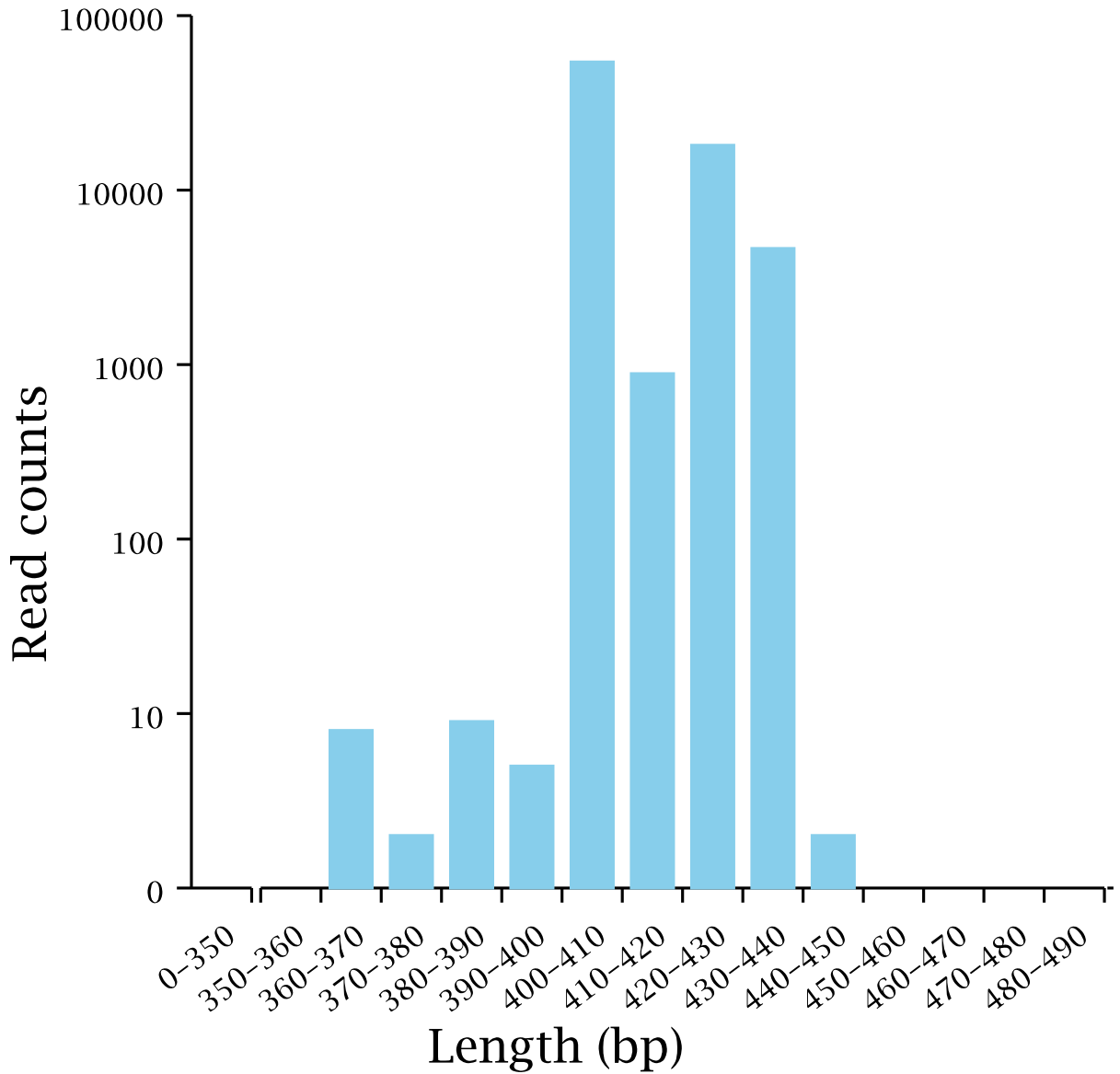

Supplement: Supplementary file 1 [file Data_Sheet_1.ZIP › Supplementary Figure S1/WA6_reads_length/WA6_reads_length.pdf]

# Length Distribution

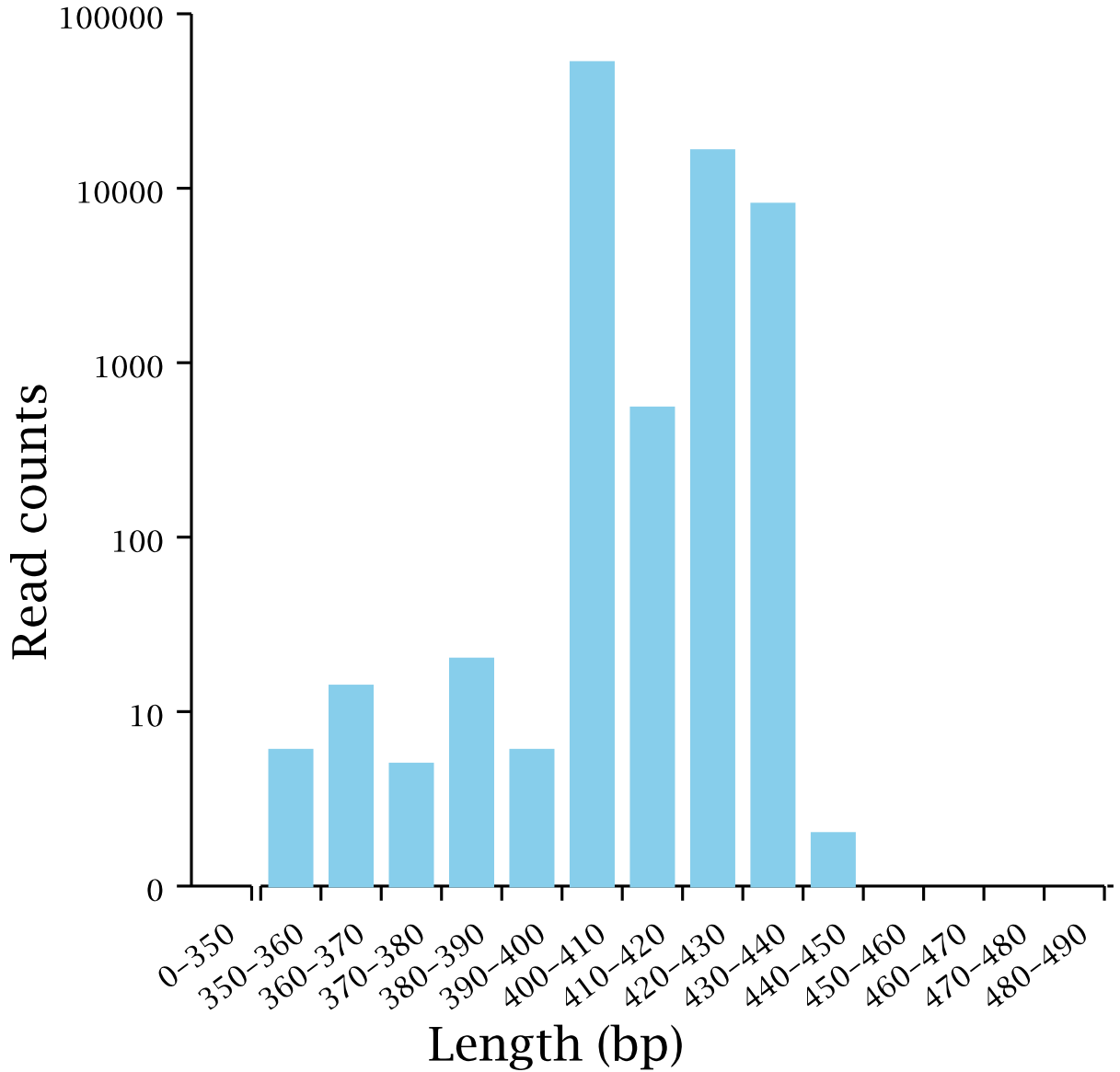

Supplement: Supplementary file 1 [file Data_Sheet_1.ZIP › Supplementary Figure S1/WJ1_reads_length/WJ1_reads_length.pdf]

# Length Distribution

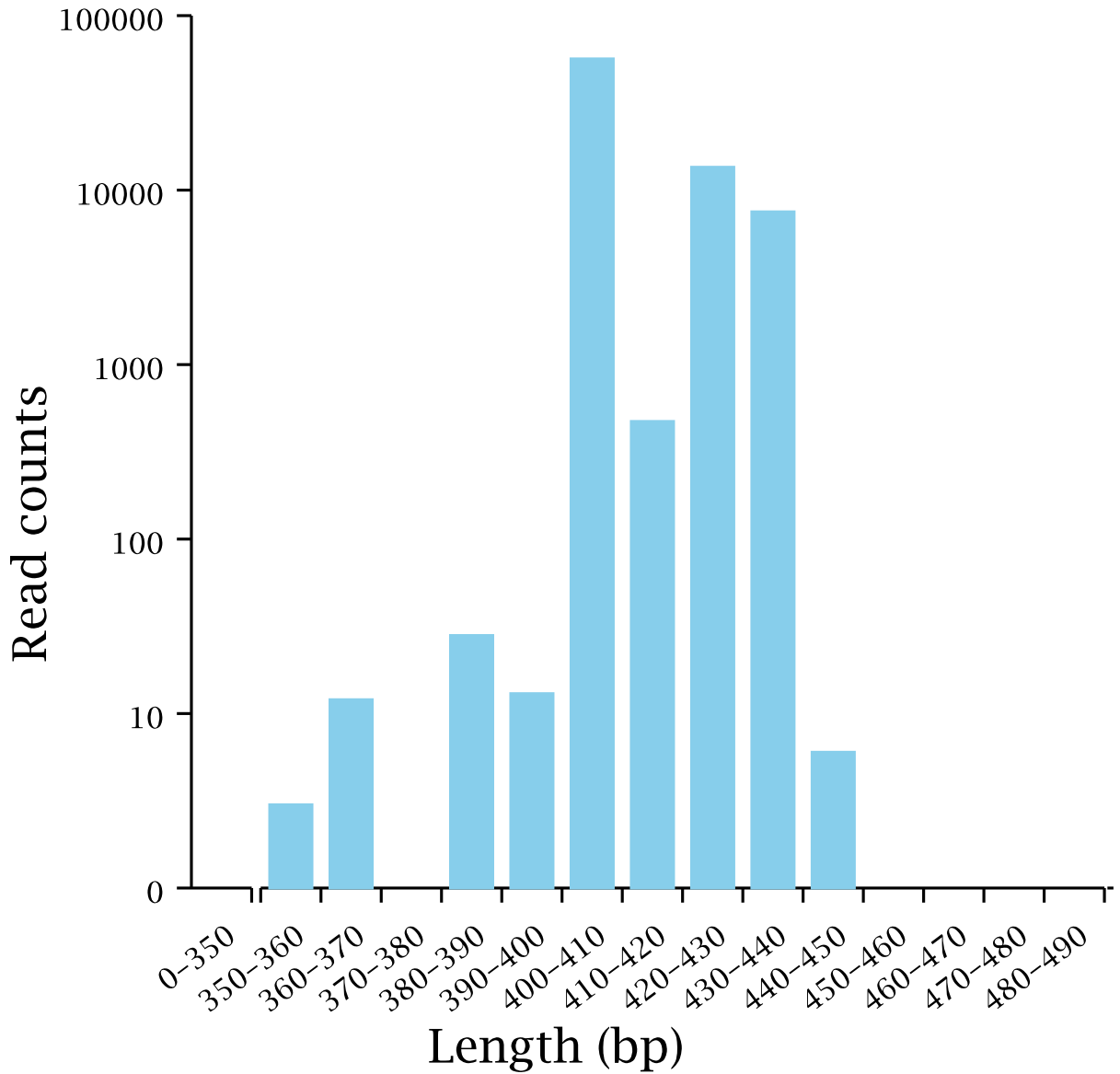

Supplement: Supplementary file 1 [file Data_Sheet_1.ZIP › Supplementary Figure S1/WJ2_reads_length/WJ2_reads_length.pdf]

# Length Distribution

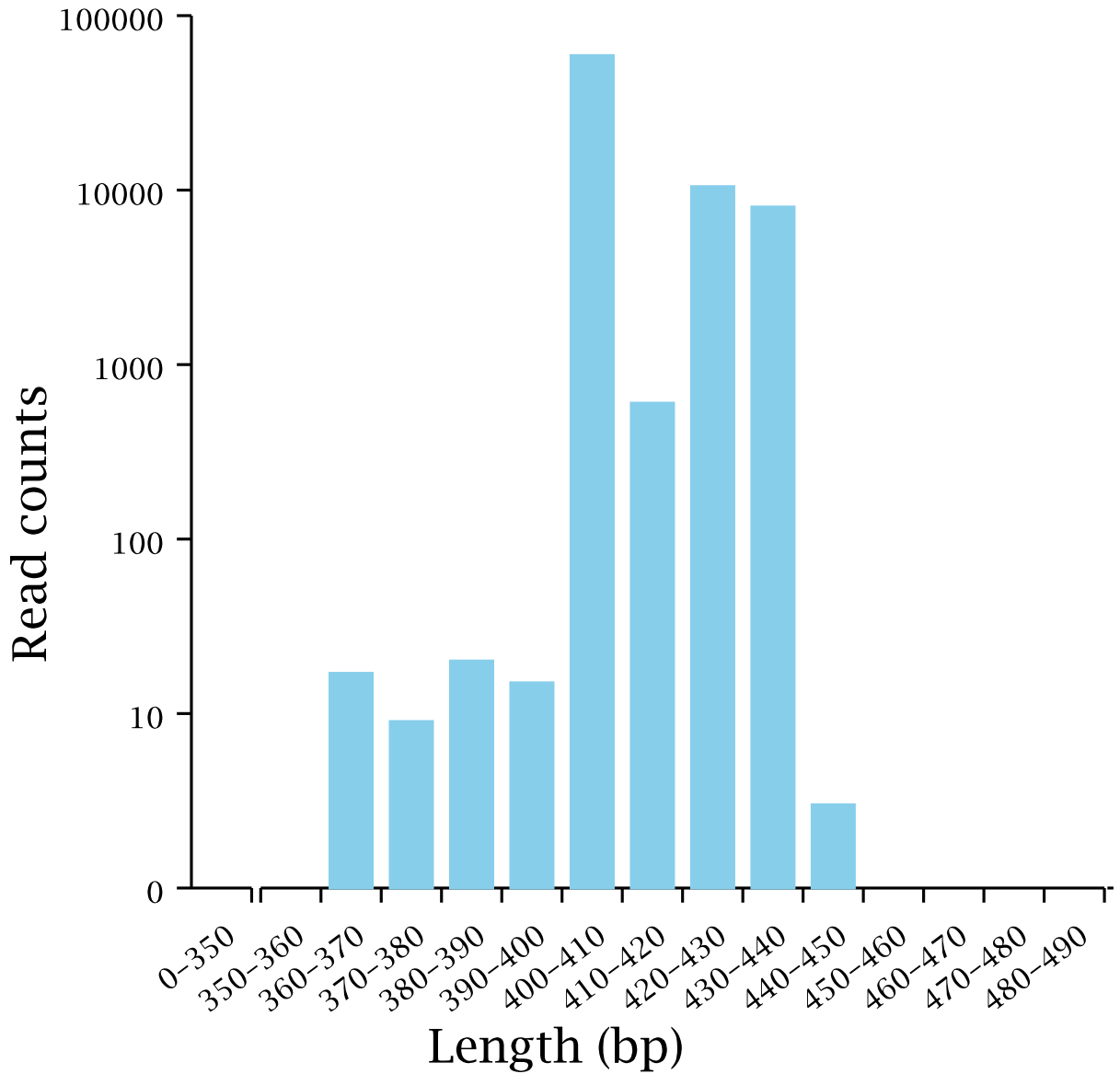

Supplement: Supplementary file 1 [file Data_Sheet_1.ZIP › Supplementary Figure S1/WJ3_reads_length/WJ3_reads_length.pdf]

# Length Distribution

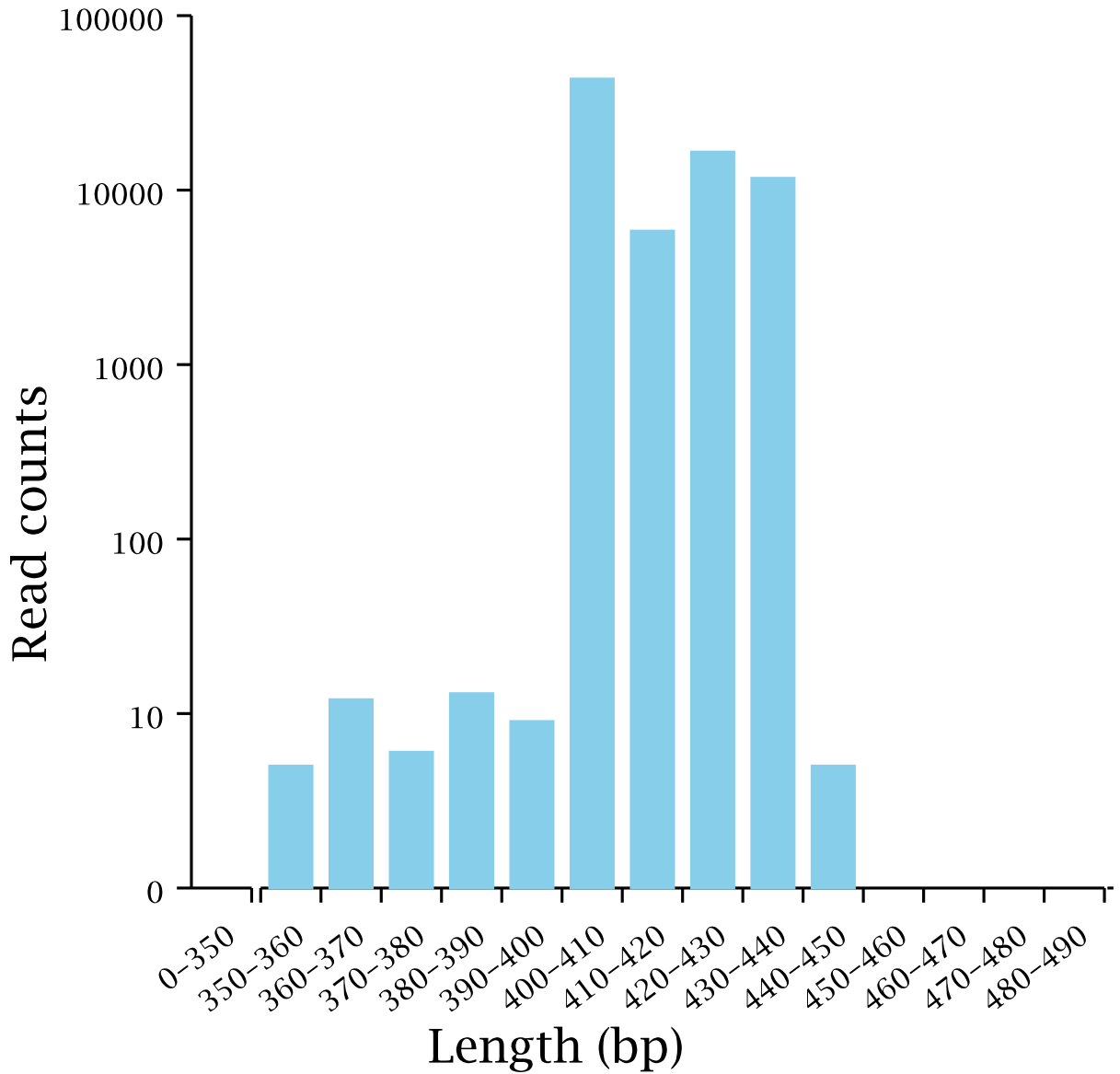

Supplement: Supplementary file 1 [file Data_Sheet_1.ZIP › Supplementary Figure S1/WJ4_reads_length/WJ4_reads_length.pdf]

# Length Distribution

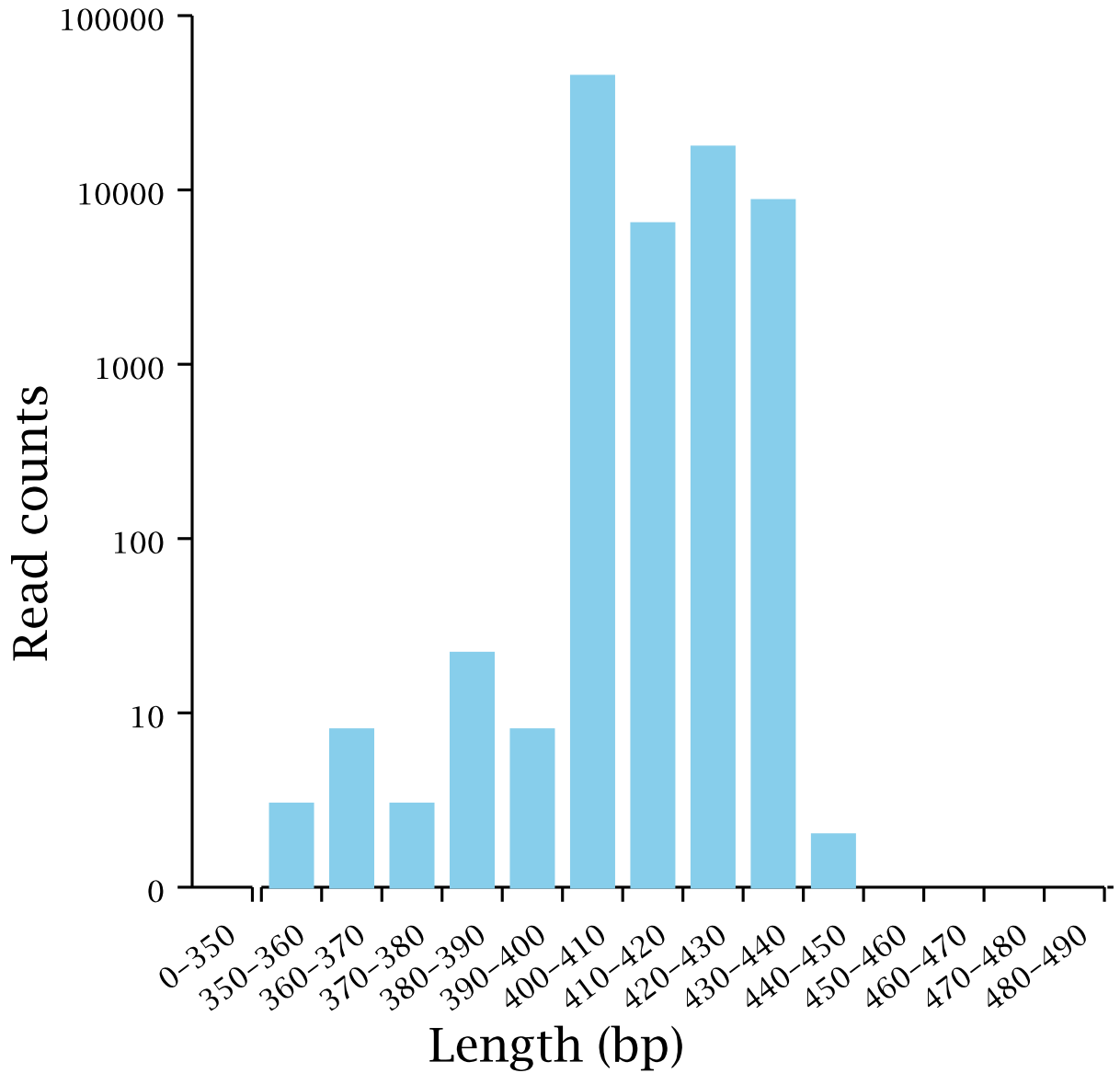

Supplement: Supplementary file 1 [file Data_Sheet_1.ZIP › Supplementary Figure S1/WJ5_reads_length/WJ5_reads_length.pdf]

# Length Distribution

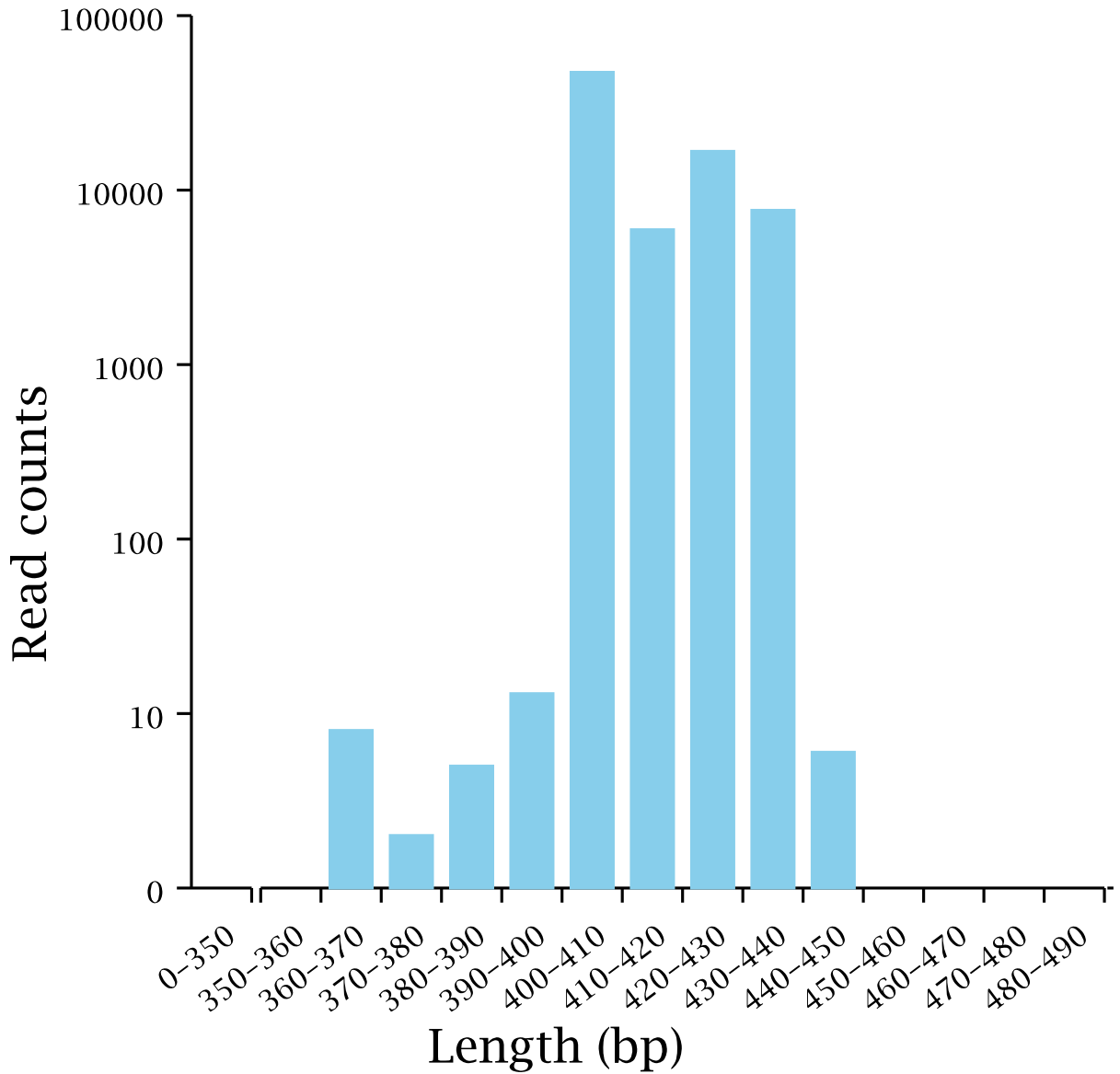

Supplement: Supplementary file 1 [file Data_Sheet_1.ZIP › Supplementary Figure S1/WJ6_reads_length/WJ6_reads_length.pdf]
