# Supplementary material for: Changes in the gut microbiota of forest musk deer (Moschus berezovskii) during ex situ conservation
Source: Front Microbiol. 2022 Sep 8;13:969593. doi: 10.3389/fmicb.2022.969593 (PMC9493438; doi:10.3389/fmicb.2022.969593)
Supplement: Supplementary file 2 [file Data_Sheet_2.ZIP › Supplementary Table/Supplementary Table S1.docx]

**Supplementary Table S1** Weinan and Huailai forest musk deer breeding base.

|  | Weinan forest musk deer breeding base | Huailai forest musk deer breeding base |
| --- | --- | --- |
| Geographic coordinates | N 34°13′ ~ 35°52′ E 108°50′ ~ 110°38′ | N 40° 4′ ~ 40°35′ E 155°6′ ~ 115°58′ |
| Annual average temperature | 13.6℃ | 9.1℃ |
| Annual average Precipitation | 600 mm | 396 mm |
| Altitude | 390 m | 1200 m |
| Population | 300 | 200 |
