# Supplementary material for: Changes in the gut microbiota of forest musk deer (Moschus berezovskii) during ex situ conservation
Source: Front Microbiol. 2022 Sep 8;13:969593. doi: 10.3389/fmicb.2022.969593 (PMC9493438; doi:10.3389/fmicb.2022.969593)
Supplement: Supplementary file 2 [file Data_Sheet_2.ZIP › Supplementary Table/Supplementary Table S2.docx]

**Supplementary Table S2** Statistics of sequencing data of each sample after filtration.

| Sample ID | Raw Reads | Clean Reads | Effective Reads | AvgLen(bp) | GC(%) | Q20(%) | Q30(%) | Effective(%) |
| --- | --- | --- | --- | --- | --- | --- | --- | --- |
| HA1 | 79915 | 79678 | 77659 | 411 | 52.20 | 99.07 | 96.20 | 97.18 |
| HA2 | 80258 | 80032 | 78222 | 411 | 52.66 | 99.11 | 96.37 | 97.46 |
| HA3 | 80270 | 80057 | 77778 | 413 | 52.55 | 99.07 | 96.23 | 96.90 |
| HA4 | 79778 | 79548 | 78096 | 411 | 52.59 | 99.08 | 96.28 | 97.89 |
| HA5 | 80250 | 80028 | 78627 | 411 | 52.86 | 99.12 | 96.37 | 97.98 |
| HA6 | 80154 | 79905 | 77898 | 412 | 52.72 | 99.10 | 96.31 | 97.19 |
| HJ1 | 80284 | 80070 | 78250 | 411 | 52.75 | 99.04 | 96.15 | 97.47 |
| HJ2 | 80301 | 80098 | 78134 | 411 | 52.80 | 99.09 | 96.31 | 97.30 |
| HJ3 | 80295 | 80084 | 78829 | 411 | 52.44 | 99.10 | 96.34 | 98.17 |
| HJ4 | 79870 | 79647 | 75720 | 413 | 52.49 | 99.08 | 96.25 | 94.80 |
| HJ5 | 80360 | 80144 | 77511 | 411 | 52.50 | 99.09 | 96.31 | 96.45 |
| HJ6 | 80054 | 79824 | 77889 | 413 | 52.43 | 99.05 | 96.16 | 97.30 |
| WA1 | 80144 | 79865 | 77911 | 412 | 52.96 | 99.02 | 96.05 | 97.21 |
| WA2 | 60690 | 60466 | 59125 | 415 | 51.72 | 98.97 | 95.86 | 97.42 |
| WA3 | 58708 | 58501 | 57294 | 410 | 52.91 | 99.05 | 96.16 | 97.59 |
| WA4 | 79794 | 79534 | 77860 | 412 | 52.67 | 99.09 | 96.27 | 97.58 |
| WA5 | 80196 | 79931 | 78287 | 410 | 52.60 | 99.13 | 96.41 | 97.62 |
| WA6 | 79906 | 79649 | 77851 | 411 | 52.74 | 99.07 | 96.19 | 97.43 |
| WJ1 | 79895 | 79599 | 77626 | 412 | 52.93 | 99.00 | 96.02 | 97.16 |
| WJ2 | 80139 | 79881 | 78059 | 411 | 53.09 | 99.05 | 96.16 | 97.40 |
| WJ3 | 80208 | 79913 | 78158 | 411 | 53.30 | 99.04 | 96.15 | 97.44 |
| WJ4 | 79845 | 79530 | 77361 | 414 | 52.79 | 98.98 | 95.94 | 96.89 |
| WJ5 | 80032 | 79729 | 77648 | 413 | 52.74 | 99.00 | 95.99 | 97.02 |
| WJ6 | 79789 | 79478 | 77618 | 413 | 52.97 | 99.01 | 96.02 | 97.28 |
