# Supplementary material for: Changes in the gut microbiota of forest musk deer (Moschus berezovskii) during ex situ conservation
Source: Front Microbiol. 2022 Sep 8;13:969593. doi: 10.3389/fmicb.2022.969593 (PMC9493438; doi:10.3389/fmicb.2022.969593)
Supplement: Supplementary file 2 [file Data_Sheet_2.ZIP › Supplementary Table/Supplementary Table S3.docx]

**Supplementary Table S3** OTU species of samples on various levels.

| Sample | Kingdom | Phylum | Class | Order | Family | Genus | Species |
| --- | --- | --- | --- | --- | --- | --- | --- |
| HA1 | 1 | 12 | 19 | 29 | 54 | 126 | 133 |
| HA2 | 1 | 14 | 21 | 31 | 56 | 136 | 144 |
| HA3 | 1 | 17 | 24 | 37 | 64 | 147 | 154 |
| HA4 | 2 | 17 | 25 | 38 | 66 | 149 | 154 |
| HA5 | 1 | 15 | 23 | 34 | 61 | 136 | 140 |
| HA6 | 2 | 15 | 22 | 35 | 61 | 141 | 147 |
| HJ1 | 2 | 16 | 24 | 35 | 66 | 147 | 153 |
| HJ2 | 1 | 15 | 22 | 34 | 61 | 143 | 147 |
| HJ3 | 2 | 16 | 22 | 33 | 61 | 141 | 148 |
| HJ4 | 2 | 15 | 22 | 35 | 63 | 139 | 144 |
| HJ5 | 2 | 15 | 22 | 31 | 55 | 132 | 138 |
| HJ6 | 2 | 17 | 24 | 35 | 60 | 145 | 152 |
| WA1 | 2 | 18 | 27 | 47 | 77 | 176 | 185 |
| WA2 | 2 | 19 | 28 | 48 | 77 | 172 | 182 |
| WA3 | 2 | 18 | 27 | 43 | 70 | 166 | 174 |
| WA4 | 2 | 18 | 27 | 50 | 78 | 173 | 181 |
| WA5 | 2 | 18 | 27 | 46 | 76 | 176 | 185 |
| WA6 | 2 | 19 | 28 | 51 | 82 | 182 | 192 |
| WJ1 | 2 | 18 | 27 | 47 | 77 | 173 | 183 |
| WJ2 | 2 | 19 | 28 | 50 | 81 | 174 | 182 |
| WJ3 | 2 | 17 | 26 | 48 | 78 | 172 | 182 |
| WJ4 | 2 | 18 | 27 | 50 | 82 | 179 | 189 |
| WJ5 | 2 | 18 | 27 | 49 | 79 | 177 | 184 |
| WJ6 | 1 | 16 | 25 | 47 | 79 | 176 | 186 |
| Total | 2 | 19 | 28 | 53 | 86 | 196 | 208 |
