# Supplementary material for: Changes in the gut microbiota of forest musk deer (Moschus berezovskii) during ex situ conservation
Source: Front Microbiol. 2022 Sep 8;13:969593. doi: 10.3389/fmicb.2022.969593 (PMC9493438; doi:10.3389/fmicb.2022.969593)
Supplement: Supplementary file 2 [file Data_Sheet_2.ZIP › Supplementary Table/Supplementary Table S4.docx]

**Supplementary Table S4** OTU clustering results of samples on various levels.

| Sample | Kingdom | Phylum | Class | Order | Family | Genus | Species |
| --- | --- | --- | --- | --- | --- | --- | --- |
| HA1 | 64157 | 64157 | 64157 | 64157 | 64157 | 64157 | 64157 |
| HA2 | 65957 | 65957 | 65957 | 65957 | 65957 | 65957 | 65957 |
| HA3 | 59864 | 59864 | 59864 | 59864 | 59864 | 59864 | 59864 |
| HA4 | 66241 | 66241 | 66241 | 66241 | 66241 | 66241 | 66241 |
| HA5 | 64302 | 64302 | 64302 | 64302 | 64302 | 64302 | 64302 |
| HA6 | 63760 | 63760 | 63760 | 63760 | 63760 | 63760 | 63760 |
| HJ1 | 63994 | 63994 | 63994 | 63994 | 63994 | 63994 | 63994 |
| HJ2 | 65250 | 65250 | 65250 | 65250 | 65250 | 65250 | 65250 |
| HJ3 | 66361 | 66361 | 66361 | 66361 | 66361 | 66361 | 66361 |
| HJ4 | 61535 | 61535 | 61535 | 61535 | 61535 | 61535 | 61535 |
| HJ5 | 64896 | 64896 | 64896 | 64896 | 64896 | 64896 | 64896 |
| HJ6 | 57725 | 57725 | 57725 | 57725 | 57725 | 57725 | 57725 |
| WA1 | 63628 | 63628 | 63628 | 63628 | 63628 | 63628 | 63628 |
| WA2 | 46884 | 46884 | 46884 | 46884 | 46884 | 46884 | 46884 |
| WA3 | 45959 | 45959 | 45959 | 45959 | 45959 | 45959 | 45959 |
| WA4 | 62326 | 62326 | 62326 | 62326 | 62326 | 62326 | 62326 |
| WA5 | 66142 | 66142 | 66142 | 66142 | 66142 | 66142 | 66142 |
| WA6 | 65940 | 65940 | 65940 | 65940 | 65940 | 65940 | 65940 |
| WJ1 | 65025 | 65025 | 65025 | 65025 | 65025 | 65025 | 65025 |
| WJ2 | 63551 | 63551 | 63551 | 63551 | 63551 | 63551 | 63551 |
| WJ3 | 65877 | 65877 | 65877 | 65877 | 65877 | 65877 | 65877 |
| WJ4 | 64143 | 64143 | 64143 | 64143 | 64143 | 64143 | 64143 |
| WJ5 | 64381 | 64381 | 64381 | 64381 | 64381 | 64381 | 64381 |
| WJ6 | 64678 | 64678 | 64678 | 64678 | 64678 | 64678 | 64678 |
